# AI-assisted hierarchical primary care and health equity among older adults with chronic diseases: a matched observational mixed-methods study in China

**DOI:** 10.3389/fpubh.2026.1909288

**Published:** 2026-09-22

**Authors:** Hang Cheng, Li Jia

**Affiliations:** School of Humanities and Management, Guangdong Medical University, Dongguan, China

**Keywords:** artificial intelligence, chronic disease management, digital health literacy, hierarchical diagnosis and treatment, medical service accessibility, primary healthcare

## Abstract

**Background:**

Population aging and the growing burden of chronic diseases have increased the need for more accessible and equitable primary healthcare. Artificial intelligence (AI)-assisted hierarchical diagnosis and treatment has been increasingly introduced into primary care to support patient guidance, triage, follow-up, referral coordination, and risk warning. However, evidence remains limited on whether AI-enabled primary care is associated with more equitable chronic disease management among older adults.

**Objective:**

This study examined the associations between AI-enabled primary care within China's hierarchical medical system and health outcomes among older adults with chronic diseases. It further assessed whether these associations differed across income, education, and digital literacy groups, and whether health information acquisition and medical service accessibility were plausible pathways linking AI-enabled care to patient outcomes.

**Methods:**

A multicenter, institution-based, matched observational mixed-methods study was conducted in Guangdong Province, China. The analytic sample included 420 older adults with chronic diseases, including 210 patients recruited from AI-enabled primary healthcare institutions and 210 from matched comparison institutions. Multivariable regression models, subgroup analyses, interaction tests, exploratory pathway analyses, and robustness checks were used to examine associations between AI exposure and chronic disease control, self-rated health, and hospitalization-related outcomes. Semi-structured interviews with 48 medical staff and institutional managers were analyzed to contextualize the quantitative findings and to better understand how AI-enabled services were implemented in routine primary care.

**Results:**

Compared with patients in comparison institutions, patients in AI-enabled institutions showed a higher chronic disease control rate, better self-rated health, and lower hospitalization risk. Adjusted regression results indicated that institutional AI access was positively associated with chronic disease control (OR = 1.69, 95% CI: 1.12–2.56) and self-rated health (β = 0.24, 95% CI: 0.08–0.39), and negatively associated with hospitalization (OR = 0.63, 95% CI: 0.39–0.99). Subgroup analyses suggested that these associations were more evident among low-income and low-education patients, whereas digital literacy remained an important boundary condition. Exploratory pathway analyses showed that health information acquisition and medical service accessibility partly explained the observed associations between AI-enabled care and patient outcomes. Qualitative findings further suggested that AI-supported routine follow-up and patient guidance, but that older adults with low digital literacy often required staff-mediated assistance to benefit from AI-enabled services.

**Conclusion:**

AI-enabled primary care within China's hierarchical medical system was associated with better chronic disease management outcomes among older adults in this matched observational study. The findings suggest that AI-enabled primary care may have equity-relevant associations when accompanied by better health information acquisition, service accessibility, follow-up, and referral coordination. These associations should not be interpreted as causal relationships. Their potential equity relevance depends on whether AI tools are integrated into primary care workflows and supported by inclusive, human-assisted service models for older adults with limited digital literacy.

## 1 Introduction

Population aging and the rising burden of chronic diseases are placing increasing pressure on primary healthcare systems. For older adults, chronic disease management is rarely limited to diagnosis or medication. It involves long-term follow-up, timely referral, health information acquisition, self-management, and continuous interaction with medical institutions. Yet these processes are not equally accessible to all patients. Older adults with lower income, limited education, weaker family support, or poor digital skills often face greater difficulty in understanding health information, choosing the appropriate level of care, completing online registration, receiving follow-up reminders, or maintaining stable disease control. In this sense, health inequality among older chronic disease patients is not only reflected in clinical outcomes but also embedded in the everyday process through which they seek, understand, and use medical services. The problem is therefore practical as well as academic: if primary care reform cannot help disadvantaged older adults navigate the healthcare system more easily, health equity may remain an abstract policy slogan rather than a lived reality.

China's hierarchical diagnosis and treatment system provides a critical setting for examining this issue. The reform is designed to strengthen first contact at the primary level, promote two-way referral, and improve the allocation of medical resources across different levels of care. For older patients with hypertension, diabetes, cardiovascular disease, and other long-term conditions, such a system should ideally reduce unnecessary hospital visits and make routine management more convenient. However, existing evidence suggests that patients' willingness to use primary care is shaped by service accessibility, drug availability, cost, referral convenience, physician trust, and health literacy (1). More recent research also indicates that the effect of hierarchical medical reform on perceived primary care quality may vary across urban and rural residents and across vulnerable patient groups (2). These findings imply that institutional reform alone may not be sufficient. The key question is how patients are guided within the system. In practice, this is the point at which equity is either improved or quietly lost.

Artificial intelligence is now entering this space and may reshape the “information–decision–service utilization” chain of primary care. AI-assisted guidance, intelligent triage, chronic disease follow-up, risk warning, and decision support can potentially reduce information asymmetry, improve patient–service matching, and support more timely referral. A recent scoping review shows that AI has been increasingly applied in primary care for diagnosis, workflow support, clinical decision-making, chronic disease management, and patient engagement, although implementation barriers remain substantial (3). From an equity perspective, this creates a meaningful possibility: AI may help older patients who lack medical knowledge or service navigation ability obtain clearer advice and more convenient access to primary care. Put simply, it may help them answer a very basic but important question: “Where should I go, and what should I do next?” However, this optimistic expectation should not be accepted too quickly. AI systems may reproduce or even amplify existing inequalities if they rely on biased data, neglect disadvantaged users, or require digital skills that some older adults do not have (4, 5). For older chronic disease patients, digital health literacy is especially important because chronic disease management increasingly depends on the ability to find, evaluate, and use online health information (6).

This tension motivates the present study. AI embedded in primary hierarchical diagnosis and treatment may be associated with either more inclusive care or new forms of digital exclusion. Whether favorable associations are observed depends not only on institutional adoption but also on actual use, information acquisition, service accessibility, and the ability of socioeconomically and digitally disadvantaged patients to engage with AI-supported care. Accordingly, this study examines associations between AI-enabled primary care and patient-reported chronic disease control, self-rated health, and hospitalization-related outcomes; variation across income, education, and digital-literacy groups; and exploratory pathways involving health information acquisition and medical service accessibility. The study asks for whom favorable associations are observed and under what implementation conditions, without treating the observational design as evidence that AI caused the outcomes.

## 2 Literature review and theoretical background

### 2.1 Health equity, aging, and chronic disease management

Health equity generally refers to the absence of avoidable, unfair, and systematic differences in health across population groups. It is not the same as health equality. Equality emphasizes the same distribution of resources or outcomes, whereas equity asks whether differences are socially produced, morally problematic, and preventable (7). For older adults, this distinction is particularly important. Aging itself does not automatically produce poor health; rather, health in later life is shaped by accumulated social, economic, educational, behavioral, and healthcare-related advantages or disadvantages over the life course. The World Health Organization has emphasized that healthy aging depends not only on medical care but also on supportive environments, social participation, and the ability to maintain functional capacity (8).

Chronic disease management is one of the most important fields in which health inequity among older adults becomes visible. Older patients with chronic diseases often need long-term medication, lifestyle adjustment, regular monitoring, follow-up visits, and timely referral. These tasks require not only clinical services but also health knowledge, care coordination, communication with providers, and self-management capacity. Recent research in China shows that chronic diseases and multimorbidity are significantly associated with limitations in activities of daily living among older adults, suggesting that chronic disease burden is closely linked with functional decline and quality of life (9). Similar studies have also highlighted that multimorbidity among older adults creates complex care needs that are difficult to address through fragmented, hospital-centered services (10).

From a theoretical perspective, this study understands health equity through the lens of social determinants of health and healthcare access. The social determinants perspective suggests that health outcomes are shaped by income, education, social support, residential environment, and institutional arrangements, rather than by individual biological factors alone. The access-to-care framework further argues that healthcare access is produced through the interaction between service supply and patient ability, including approachability, acceptability, availability, affordability, and appropriateness of services, as well as patients' ability to perceive, seek, reach, pay, and engage with care (11). This framework is especially useful for studying older chronic disease patients because their health outcomes depend on whether they can recognize health needs, obtain understandable information, reach appropriate providers, and remain engaged in long-term management.

Therefore, health equity in chronic disease management should not be measured only by final health outcomes, such as blood pressure control, blood glucose control, hospitalization, or self-rated health. It should also be examined through the care process that leads to these outcomes. For example, two older patients may both have hypertension, but one may know how to monitor blood pressure, receive regular follow-up reminders, understand when referral is needed, and communicate smoothly with primary care providers, while the other may not. Their clinical gap may begin as an information gap, a navigation gap, or an access gap. This logic provides the theoretical basis for this study: artificial intelligence embedded in primary care may influence health equity by changing the process through which older chronic disease patients acquire information, make care decisions, and use medical services.

### 2.2 Hierarchical diagnosis and treatment, and primary care in China

China's hierarchical diagnosis and treatment system was introduced to address the long-standing imbalance between overcrowded tertiary hospitals and relatively underused primary healthcare institutions. The reform aims to encourage first-contact care at the primary level, establish two-way referral, separate acute and chronic care needs, and strengthen coordination between different levels of medical institutions. In principle, this system is highly relevant to chronic disease management because many chronic conditions do not require frequent treatment in tertiary hospitals. Instead, they require continuous monitoring, stable medication management, health education, follow-up, and early intervention, all of which are functions that primary healthcare institutions are expected to provide.

However, the implementation of hierarchical diagnosis and treatment is not merely a matter of institutional design. It also depends on patient trust, provider capacity, service quality, referral convenience, drug availability, and local health system coordination. Luo et al. (1), based on a large empirical study of chronic disease patients in Tianjin, found that patients' preferences for primary care under China's hierarchical medical system are influenced by physician service quality, referral convenience, treatment standards, and health literacy. Their findings suggest that patients do not automatically choose primary care simply because the policy encourages them to do so. They need to believe that primary care is reliable, convenient, and capable of solving their problems.

Recent evidence also indicates that the effect of the hierarchical medical system may vary across groups. Zhao et al. (2) found that the implementation of China's hierarchical medical system had limited effects on improving perceived primary care quality, especially among rural residents and patients with chronic diseases. This finding is important because it suggests that the reform may not equally benefit all population groups. Similarly, Wang and Chen (12) found that the health effects of the hierarchical medical system differ according to health insurance participation and literacy level. These studies collectively indicate that hierarchical diagnosis and treatment have an equity dimension: it may improve service allocation in general, but its benefits may be unevenly distributed if vulnerable groups face greater barriers in using primary care.

The theoretical implication is that primary care is not only a service site but also a coordination platform. For older chronic disease patients, primary care should help them understand their condition, decide whether they need referral, receive follow-up, and avoid unnecessary hospitalization. In Andersen's behavioral model of health service utilization, medical service use is shaped by pre-disposing characteristics, enabling resources, and need factors (13). Applied to this study, age, gender, education, income, insurance status, chronic disease type, multimorbidity, and distance to primary institutions may all influence patients' use of primary care. Yet the hierarchical system itself also acts as an enabling or constraining structure. If the system provides clear guidance and accessible services, it may reduce inequality. If it is difficult to navigate, it may reinforce existing disparities.

This is where artificial intelligence becomes theoretically meaningful. AI-assisted triage, intelligent guidance, risk warning, and follow-up reminders may strengthen the coordinating function of primary care. These tools may help patients identify the appropriate level of care, reduce uncertainty in referral decisions, and improve service continuity. However, whether AI can actually improve health equity within the hierarchical system remains an empirical question. Existing studies on hierarchical diagnosis and treatment have rarely examined AI as a factor that may reshape the relationship between institutional reform, patient behavior, and health outcomes.

### 2.3 Artificial intelligence in primary care and chronic disease services

Artificial intelligence has increasingly been introduced into healthcare systems for diagnosis, prediction, decision support, workflow optimization, patient engagement, and chronic disease management. In primary care, AI has particular potential because primary healthcare institutions face multiple pressures, including workforce shortages, increasing multimorbidity, high administrative burden, and the need to provide continuous care for large populations. A recent scoping review by Katonai and Arvai (3) found that AI applications in primary healthcare can be grouped into early intervention and decision support, chronic disease management, operations and patient management, and implementation experiences. The review also noted that although AI tools often show technical promise, their routine implementation is limited by workflow misalignment, usability barriers, trust concerns, equity gaps, and financial constraints.

In chronic disease services, AI may support both providers and patients. For providers, AI can assist in risk stratification, follow-up prioritization, medication management, complication prediction, and clinical decision support. For patients, AI-enabled tools may provide self-management support, personalized health education, symptom monitoring, lifestyle recommendations, and reminders. Hwang et al. (14) reviewed AI applications for chronic condition self-management and found that AI may support medical, behavioral, and emotional self-management tasks. This is relevant to older chronic disease patients because disease control depends not only on clinical treatment but also on everyday self-care, medication adherence, diet, exercise, and timely response to warning signs.

Nevertheless, the literature also shows that AI in primary care is not automatically beneficial. Abbasgholizadeh Rahimi et al. (15) pointed out that although AI in community-based primary healthcare may facilitate diagnosis and disease management, there are concerns about unintended harmful effects, limited real-world evaluation, and insufficient integration into community care contexts. More recent work has further emphasized that AI tools should be assessed not only by accuracy but also by implementation feasibility, explainability, acceptability, and alignment with clinical workflow (3). For primary care, this point is crucial. A technically accurate AI system may still fail if doctors do not trust it, patients cannot use it, or institutions do not have the capacity to integrate it into daily service routines.

The equity implications of AI are even more complex. d'Elia et al. (16) argued that AI may affect health inequities in primary care both through direct care provision and through broader system-level effects. On the positive side, AI may improve access, reduce waiting time, support triage, and help allocate resources more efficiently. On the negative side, AI may reproduce bias if training data underrepresent disadvantaged groups, if algorithms perform differently across socioeconomic groups, or if implementation favors digitally capable users. Sasseville et al. (5) similarly noted that primary care AI models may perpetuate or amplify existing biases toward diverse groups, and that bias mitigation requires attention to protected attributes, data practices, and stakeholder involvement.

For this study, AI-assisted hierarchical diagnosis and treatment are conceptualized as a sociotechnical arrangement rather than a purely technical variable. Its observed associations may depend on interactions among algorithmic systems, primary care institutions, providers, and patients. Favorable health equity-related associations are plausible when AI reduces information asymmetry, supports navigation, and makes primary care more accessible, whereas unfavorable associations are plausible when implementation requires digital capabilities that some older adults do not possess. The empirical analysis, therefore, treats AI as an institutional and individual exposure embedded in real care processes, not as an isolated causal intervention.

### 2.4 Digital divide, digital health literacy, and older adults

The digital divide is a key boundary condition for understanding whether AI-enabled primary care can promote health equity. In early studies, the digital divide mainly referred to unequal access to digital devices and the internet. However, in contemporary digital health research, the concept has expanded to include differences in digital skills, usage patterns, information evaluation, confidence, trust, and the ability to translate digital information into health behavior. This shift is important because many older adults may own smartphones but still have difficulty using online registration systems, health apps, AI guidance tools, or digital follow-up platforms independently.

Digital health literacy refers to the ability to seek, find, understand, appraise, and apply health information from digital sources to solve health problems. For older adults with chronic diseases, digital health literacy is directly related to chronic disease management because many health services are now mediated through digital platforms. Zaghloul et al. (6), in a systematic review and meta-analysis of patients with diabetes, hypertension, and rheumatoid arthritis, found that digital health literacy is shaped by demographic and socioeconomic factors and is relevant to disease management. This means that digital health literacy is not simply an individual skill; it is also socially distributed.

Recent studies on older adults have provided stronger evidence for this point. Yang and Gao (17) found that the digital divide remains persistent among older adults and is associated with self-rated health, especially among socially disadvantaged groups with lower income and education. Li and Ouyang (18), using nationally representative data from the China Health and Retirement Longitudinal Study, showed that although digital access and usage divides among Chinese older adults declined from 2011 to 2020, digital divides still had negative effects on healthy aging. These findings are directly relevant to the present study because they suggest that digital inclusion is not a secondary issue. It may shape whether older adults can benefit from digital and AI-enabled health services.

For older adults with chronic diseases, digital health adoption is influenced by both personal and contextual factors. Bertolazzi and Quagila (19) showed that health technology adoption among older adults with chronic diseases is shaped by perceived usefulness, usability, social support, health needs, trust, and barriers related to age, education, and physical limitations. Hepburn and Williams (20) further emphasized that digital health adoption among older adults with chronic diseases is affected by capability, opportunity, and motivation, and that rural–urban differences and equity-relevant factors should be considered. Evidence from China also shows that older chronic disease patients often face cognitive blind spots, limited ability to evaluate digital health information, anxiety about privacy and fraud, and reliance on family members or familiar social networks for digital assistance (21).

These findings indicate that digital literacy may moderate the association between AI-assisted hierarchical diagnosis and treatment and patient outcomes. Higher digital literacy may be associated with more convenient use and lower information costs, whereas patients with lower digital literacy may rely on offline consultation, family support, or traditional information channels. The equity relevance of AI, therefore, cannot be inferred from institutional adoption alone; actual use and differences between high- and low-digital-literacy groups must also be examined.

### 2.5 Research gaps

Although the existing literature has provided valuable insights into health equity, aging, chronic disease management, China's hierarchical diagnosis and treatment system, AI-enabled primary care, and digital health literacy, several gaps remain. First, studies on older adults with chronic diseases have mainly examined how socioeconomic status, multimorbidity, health literacy, and access to care influence health outcomes, while less attention has been paid to how new digital and AI-enabled service arrangements may reshape these inequalities within primary care. Second, research on China's hierarchical diagnosis and treatment system has largely focused on patient preferences, primary care quality, service utilization, referral behavior, and resource allocation. However, the role of AI-assisted guidance, triage, follow-up, and risk warning in changing the operation of hierarchical care at the grassroots level has not been sufficiently examined. Third, while studies on AI in primary care have expanded rapidly, much of the discussion still centers on technical performance, clinical decision support, implementation feasibility, and provider acceptance. The distributional consequences of AI—especially whether it narrows or widens health gaps among vulnerable older patients—remain underexplored in real-world primary care settings.

In addition, the proposed pathways and boundary conditions linking AI-assisted hierarchical diagnosis and treatment with health equity remain insufficiently specified. Health information acquisition and medical service accessibility may form plausible intermediate pathways, while digital literacy may condition the observed associations. The present study, therefore, examines adjusted associations between AI availability or use intensity and health outcomes, heterogeneity across socioeconomic and digital-literacy groups, and exploratory cross-sectional pathways through information and accessibility.

## 3 Theoretical framework and research hypotheses

### 3.1 AI-assisted hierarchical diagnosis and treatment and health outcomes

This study conceptualizes AI-assisted hierarchical diagnosis and treatment as a sociotechnical arrangement embedded in primary healthcare rather than as a stand-alone digital tool. In the context of China's hierarchical medical system, AI may support intelligent guidance, assisted triage, chronic disease follow-up, risk warning, and referral decision-making. These functions may influence older chronic disease patients by changing how they enter the healthcare system, how they understand their conditions, and how they are matched with appropriate medical services. The theoretical basis of this relationship can be understood through the access-to-care framework and the behavioral model of health service utilization. The access-to-care framework emphasizes that healthcare access is produced through the interaction between service characteristics and patients' abilities to perceive, seek, reach, pay for, and engage with healthcare (11). Andersen's behavioral model similarly suggests that healthcare utilization is shaped by pre-disposing characteristics, enabling resources, and health needs (13). AI-assisted hierarchical diagnosis and treatment may function as a new enabling resource. It can reduce uncertainty in care-seeking, help patients identify suitable service levels, and support more continuous chronic disease management.

Recent research on AI in primary care suggests that AI has the potential to support diagnosis, workflow efficiency, clinical decision-making, chronic disease management, and patient engagement, although its implementation depends on workflow integration, trust, usability, and institutional readiness (3). For older adults with chronic diseases, these AI-supported functions may contribute to better chronic disease control, improved self-rated health, and reduced avoidable hospitalization by improving follow-up, early warning, and service matching. Accordingly, this study proposes:

**H1:** AI-assisted hierarchical diagnosis and treatment is positively associated with better health outcomes among older adults with chronic diseases, including improved chronic disease control, better self-rated health, and reduced hospitalization risk.

### 3.2 Health information acquisition mechanism

Health information acquisition is a proposed pathway linking AI-assisted hierarchical diagnosis and treatment with health outcomes. Older patients need continuing information about medication, symptoms, lifestyle management, follow-up, and referral. Because information access is socially patterned, the analysis examines whether institutional AI availability is associated with patients' reported access to timely, understandable, and actionable information ((14)).

AI-assisted hierarchical diagnosis and treatment may be associated with health information acquisition through intelligent guidance, follow-up reminders, risk-warning messages, and personalized health education. These functions provide a theoretical basis for an indirect-association pathway, but the cross-sectional design does not establish that AI exposure preceded changes in information acquisition or that those changes subsequently produced the outcomes. This leads to the following hypotheses:

**H2a:** AI-assisted hierarchical diagnosis and treatment is positively associated with health information acquisition among older adults with chronic diseases.
**H2b:** health information acquisition may help explain the relationship between AI-assisted hierarchical diagnosis and treatment and health outcomes.

### 3.3 Medical service accessibility mechanism

Medical service accessibility is another proposed pathway linking AI-assisted hierarchical diagnosis and treatment with health outcomes. Accessibility includes appointment completion, timely consultation, regular follow-up, drug access, referral procedures, and movement between levels of care. The study examines whether institutional AI availability is associated with these perceived dimensions of access rather than assuming that technology itself improves accessibility (1, 5).

AI-assisted hierarchical diagnosis and treatment may be associated with accessibility through assisted triage, risk identification, referral support, and follow-up reminders. These associations depend on whether AI recommendations are understood, trusted, connected with referral pathways, and incorporated into staff workflows. The accessibility pathway is therefore tested empirically as an exploratory cross-sectional association.

**H3a:** AI-assisted hierarchical diagnosis and treatment is positively associated with medical service accessibility among older adults with chronic diseases.
**H3b:** medical service accessibility may help explain the relationship between AI-assisted hierarchical diagnosis and treatment and health outcomes.

### 3.4 Heterogeneous effects by income, education, and digital literacy

The association between AI-assisted hierarchical diagnosis and treatment and health outcomes may vary across social groups. If favorable associations are concentrated among advantaged patients, AI-enabled services may coincide with wider inequality; if they are stronger among disadvantaged patients, the pattern may be consistent with a potentially compensatory role. Income, education, and digital literacy are therefore treated as sources of heterogeneity rather than as evidence that AI causally changed health gaps.

Education may also condition the observed association. Older adults with more education may find digital guidance easier to interpret, whereas those with limited education may require provider explanation and simplified interfaces. Whether favorable associations are observed across education groups, therefore, depends on implementation and human support.

Digital literacy is likely to be the most direct moderator. Older adults differ greatly in their ability to use smartphones, WeChat, online appointment systems, health apps, internet hospitals, and AI-based guidance tools. Recent studies show that digital health literacy among older chronic disease patients is influenced by individual capability, social support, perceived usefulness, trust, privacy concerns, and disease-related needs (6, 21). Digital divide research also suggests that digital exclusion is associated with health disadvantage among older adults (17). These findings imply that AI-enabled primary care may generate unequal benefits if low-digital-literacy patients cannot access or use the system effectively.

Digital literacy may be related both to individual AI use and to the proposed information and accessibility pathways. Patients with higher digital literacy may engage more readily with AI-supported information and services, while those with lower digital literacy may depend on clinicians, community workers, or family members. The subgroup analysis, therefore, evaluates variation in adjusted associations and does not interpret digital literacy as establishing a causal moderation process.

**H4a:** the association between AI-assisted hierarchical diagnosis and treatment and health outcomes differs across income groups.
**H4b:** the association between AI-assisted hierarchical diagnosis and treatment and health outcomes differs across education groups.
**H4c:** the association between AI-assisted hierarchical diagnosis and treatment and health outcomes differs across digital literacy groups.

### 3.5 Conceptual model

Based on the above theoretical discussion, this study specifies a conceptual model linking AI-assisted hierarchical diagnosis and treatment, health information acquisition, medical service accessibility, health outcomes, and group heterogeneity (Figure 1). Institutional AI availability and patient-level AI use intensity are treated as related but distinct exposure indicators. Health information acquisition and medical service accessibility are treated as proposed intermediate variables in an exploratory cross-sectional pathway analysis. Income, education, digital literacy, disease type, and urban-rural residence are used to examine whether the observed associations vary across patient groups.

**Figure 1:**
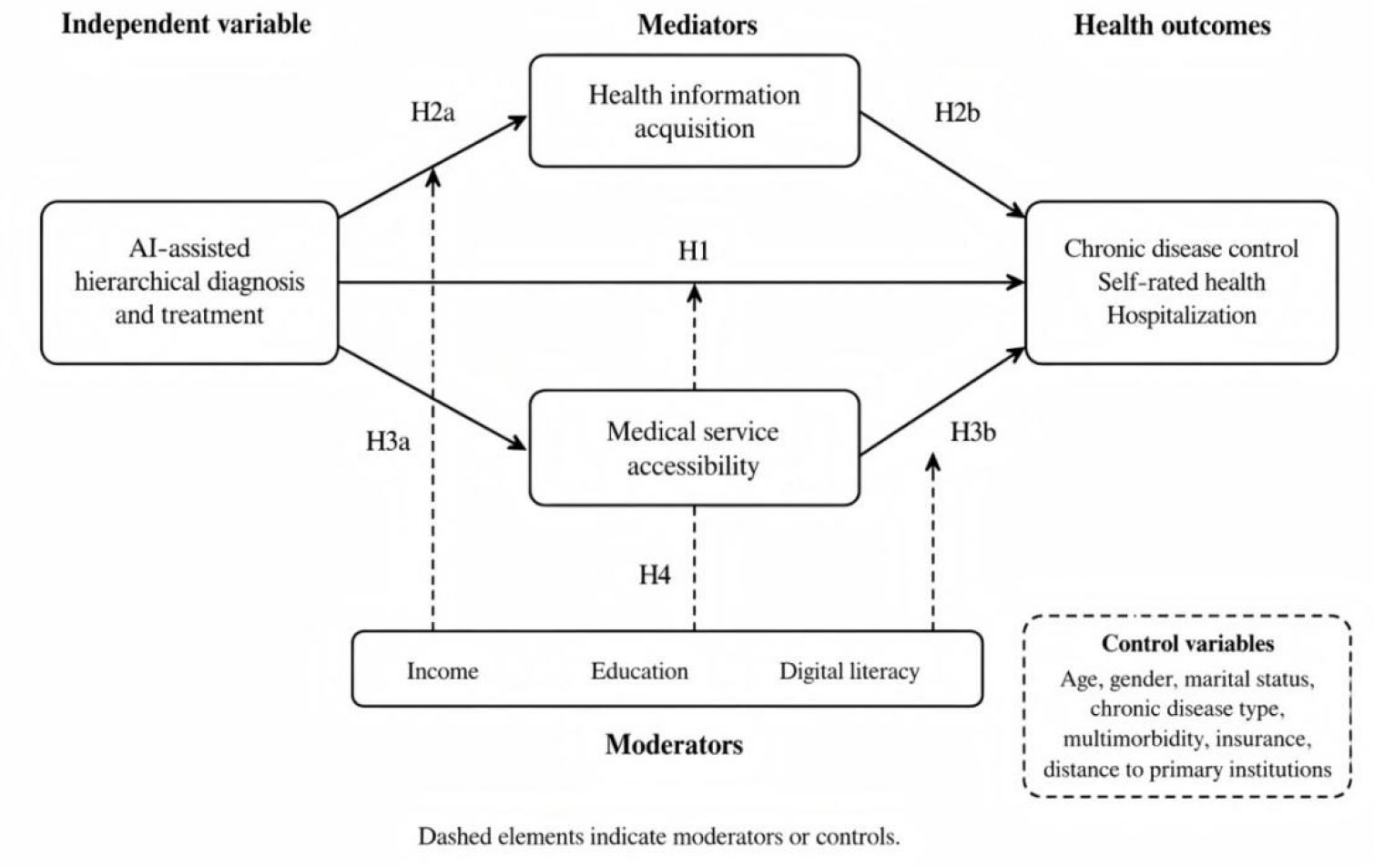
Conceptual framework.

The model proposes that institutional AI availability and individual AI use may be associated with health outcomes through both direct and indirect statistical pathways. The proposed indirect pathways involve health information acquisition and medical service accessibility. Because the exposure, proposed intermediate variables, and outcomes were measured within an observational and pre-dominantly cross-sectional framework, the model represents hypothesized associations rather than an identified causal sequence.

## 4 Research design

### 4.1 Study sites and institutional selection

This study adopted a multicenter, institution-based, matched observational mixed-methods design to examine the associations between AI-assisted hierarchical diagnosis and treatment and health equity-related outcomes among older adults with chronic diseases. The study was coordinated by Guangdong Medical University and conducted in collaboration with primary healthcare institutions, community health service centers, township hospitals, and hospital-affiliated primary care units in Guangdong Province, China. Because AI-assisted hierarchical diagnosis and treatment had already been implemented at the institutional level before the study and could not be randomly assigned to individual patients, a randomized controlled trial was not feasible. Therefore, this study used a matched comparative observational design, comparing patients from AI-enabled primary healthcare institutions with patients from comparable non-AI institutions. The quantitative component was designed and reported with reference to the STROBE statement for observational studies, while the qualitative interview component was guided by the COREQ checklist for interviews and focus groups and by mixed-methods reporting principles (22–24).

Data collection was conducted between March 2025 and December 2025. Before formal fieldwork, the research team contacted candidate institutions, verified their chronic disease management capacity, confirmed whether AI-assisted hierarchical diagnosis and treatment functions were routinely used, and assessed the feasibility of patient recruitment and staff interviews. A total of 12 primary healthcare institutions were initially assessed for eligibility. After institutional screening, eight institutions were included in the formal study, including four AI-enabled institutions and four matched comparison institutions. To protect institutional confidentiality, the four AI-enabled institutions were anonymized as PHC-A1 to PHC-A4, and the four comparison institutions were anonymized as PHC-C1 to PHC-C4. The matching pairs were coded as Pair 1 to Pair 4.

The AI-enabled institutions had routinely used at least one AI-assisted hierarchical diagnosis and treatment function for no less than 6 months before data collection. These functions included intelligent guidance, AI-assisted triage, AI-supported referral recommendation, chronic disease follow-up reminders, risk warning, and decision-support tools for primary care physicians. The specific AI systems were recorded at the institutional level, including system name, deployment time, main functional modules, target users, and service workflow. The comparison institutions provided conventional chronic disease management services but had not deployed structured AI-assisted hierarchical diagnosis and treatment during the study period.

Institutional selection followed a purposive matched sampling strategy. For each AI-enabled institution, one comparison institution was selected from the same city or from a socioeconomically comparable district. The matching criteria included service population size, urban/rural location, annual outpatient volume, number of primary care physicians, chronic disease management workload, availability of family doctor services, distance to secondary or tertiary hospitals, and local socioeconomic context. This design was used to reduce institutional-level confounding and improve comparability between the AI-enabled and comparison groups.

Healthcare institutions were included if they met the following criteria: (1) they were officially registered primary healthcare institutions, community health service centers, township hospitals, or hospital-affiliated primary care units; (2) they provided routine management services for common chronic diseases, including hypertension, diabetes, cardiovascular disease, chronic respiratory disease, or stroke-related conditions; (3) they maintained basic chronic disease follow-up records or patient management records; (4) they agreed to support questionnaire administration and medical staff interviews; and (5) they designated a local coordinator to assist with fieldwork. Institutions were excluded if they only provided specialized services without general primary care functions, if their AI system had been installed but was not used in routine care, if they were undergoing relocation or major service disruption, or if they were unable to support participant recruitment and data quality control. Of the 12 institutions assessed, four were excluded: two due to unmatched catchment areas, one due to insufficient chronic disease management records, and one due to unavailable institutional cooperation.

### 4.2 Survey sample and interview sample

The quantitative survey targeted older adults with chronic diseases who received services from the eight selected primary healthcare institutions. A total of 520 older chronic disease patients were screened. Among them, 40 were excluded during eligibility screening, including 12 who were younger than 60 years, 16 without a physician-confirmed chronic disease, and 12 with severe cognitive or communication impairment that prevented valid questionnaire completion. Therefore, 480 eligible patients were invited to participate. Of these, 40 were not enrolled, including 28 who refused participation, seven who were unable to provide informed consent, and five who withdrew before completing the survey. A total of 440 patients completed the questionnaire, corresponding to a questionnaire completion rate of 91.7% among the eligible invited patients. After data cleaning, 20 questionnaires were excluded from analysis because of missing key variables, duplicate records, or logically inconsistent responses. The final analytic sample included 420 older adults with chronic diseases, with 210 patients from AI-enabled institutions and 210 patients from comparison institutions. The final valid response rate was 87.5% among eligible invited patients.

Patient recruitment used consecutive sampling within each institution. On selected survey days, trained investigators approached potentially eligible patients after outpatient consultation, chronic disease follow-up, medication refill, health education, or family doctor service encounters. Investigators first conducted eligibility screening, explained the study purpose, obtained informed consent, and then administered the questionnaire. Consecutive sampling was adopted because it is practical in primary care fieldwork and helps reduce selective recruitment when all eligible patients during a defined recruitment period are approached. To reduce time-period bias, recruitment was conducted across different weekdays and different clinic periods rather than on a single clinic day.

Each patient was assigned a unique, anonymous study identification code at enrollment. The code consisted of the institution code and a sequential participant number. For example, patients from AI-enabled institutions were coded as PHC-A1-P001 to PHC-A4-Pxxx, and patients from comparison institutions were coded as PHC-C1-P001 to PHC-C4-Pxxx. The identification code was used to link questionnaire data, eligibility screening records, and, where applicable, de-identified clinical abstraction records. Personal identifiers such as names, phone numbers, national identification numbers, and medical record numbers were not included in the analytic dataset.

The inclusion criteria for patient participants were as follows: (1) aged 60 years or above; (2) diagnosed by a physician with at least one chronic disease requiring long-term management, such as hypertension, diabetes, cardiovascular disease, chronic respiratory disease, stroke sequelae, chronic kidney disease, or other chronic conditions; (3) having used services in the selected primary healthcare institution at least once within the past 6 months; (4) able to understand the study information and provide informed consent independently or with appropriate assistance; and (5) willing to complete the questionnaire. The exclusion criteria were: (1) severe cognitive impairment, dementia, psychiatric disorder, or communication impairment that prevented valid questionnaire completion; (2) acute medical emergency or clinically unstable condition at the time of recruitment; (3) temporary visitors who did not usually receive chronic disease services from the selected institution; (4) repeated participation across institutions; and (5) refusal or withdrawal of informed consent.

For older adults with limited literacy, visual difficulty, or difficulty completing electronic questionnaires, trained investigators provided face-to-face assisted administration using standardized wording. Family members were allowed to provide communication support when necessary, but subjective responses were required to reflect the patient's own views. This procedure was intended to reduce exclusion of older adults with lower literacy or weaker digital skills while maintaining the validity of patient-reported information.

The qualitative component included semi-structured interviews with 48 medical staff members and institutional managers, with six respondents recruited from each of the eight institutions. This produced 24 respondents from AI-enabled institutions and 24 from comparison institutions. The respondent pool represented general practitioners, nurses, public health physicians, chronic disease managers, family doctor team members, triage and referral coordinators, AI-system operators, and institutional administrators. Purposive role-based sampling was used to include both frontline and managerial perspectives on chronic disease management, referral coordination, and AI-related workflows. Short-term interns, temporary trainees, and staff without direct knowledge of these processes were excluded.

### 4.3 Questionnaire design

The patient questionnaire was developed based on the theoretical framework of this study and prior literature on healthcare access, chronic disease management, health equity, digital health literacy, and AI-enabled primary care. The questionnaire covered six domains: sociodemographic characteristics, health status and chronic disease profile, digital literacy, AI exposure and use, health information acquisition, and medical service accessibility and health outcomes.

The questionnaire development process included expert consultation, cognitive interviewing, and pilot testing. First, seven experts in primary care, public health, chronic disease management, medical informatics, gerontology, and health services research were invited to review the questionnaire for content validity. Expert review focused on whether the items adequately reflected the core constructs, whether the wording was appropriate for older adults, and whether the instrument was suitable for primary healthcare fieldwork. This process was consistent with the principle that content validity is a core measurement property of patient-reported instruments and should be established before formal psychometric testing (25). Second, cognitive interviews were conducted with 15 older chronic disease patients to assess item comprehension, response burden, cultural appropriateness, and potential ambiguity. Third, a pilot survey was conducted among 40 older chronic disease patients in one primary healthcare institution that was not included in the formal sample. Items with ambiguous wording, high non-response, severe ceiling or floor effects, or poor internal consistency were revised before formal data collection.

The study used four data sources. First, trained investigators administered paper-based or electronic patient questionnaires. Second, participating institutions supplied records of AI deployment, implementation duration, functional modules, target users, and service workflows. Third, de-identified chronic disease management or follow-up records were reviewed, where access and consent permitted, to verify diagnosed disease status. Objective blood pressure, fasting blood glucose, and HbA1c values were not sufficiently complete and harmonized across the eight institutions to support a valid record-based sensitivity analysis in the present dataset; the analysis, therefore, identifies the corresponding control outcome as patient-reported. Fourth, semi-structured interviews with medical staff and institutional managers provided qualitative contextual evidence.

Most perception-based items used a 5-point Likert scale, while demographic information, chronic disease status, AI exposure, healthcare utilization, and hospitalization were measured using categorical, binary, or count variables. The questionnaire was designed for both paper-based and electronic administration. For older participants, paper questionnaires used a large font, clear spacing, and a simplified layout. If electronic questionnaires were used, each participant was assigned a unique study ID to prevent duplicate submission. Completeness checks were used to reduce missing key variables, and the electronic survey process followed relevant principles of web-based survey reporting, including transparent description of recruitment, completion, and duplicate prevention procedures (26).

### 4.4 Variable measurement

The core exposure was AI-assisted hierarchical diagnosis and treatment, measured at two analytically distinct levels. Institutional AI availability was coded as a binary indicator of whether the institution had routinely implemented AI-assisted guidance, triage, referral support, chronic disease follow-up, risk warning, or decision-support functions for at least 6 months. Individual AI use was based on each patient's self-reported exposure to and frequency of using AI-assisted services during the previous 6 months. The primary models used institutional availability, whereas individual use and use intensity were analyzed as secondary exposure definitions because actual use may reflect patient motivation, family assistance, digital literacy, and health-seeking behavior. In descriptive analyses, 166 of 210 patients in AI-enabled institutions (79.0%) reported actual use, while 44 (21.0%) did not.

The dependent variables were patient-reported chronic disease control, self-rated health, and hospitalization-related outcomes. Patient-reported chronic disease control referred to self-reported blood pressure control among participants with hypertension and self-reported blood glucose control among participants with diabetes. The combined control indicator was retained as a summary outcome, while disease-specific subgroup models were specified separately for hypertension and diabetes to preserve clinical specificity. Self-rated health was measured on a five-point scale from very poor to very good. Hospitalization was measured as whether the patient reported a chronic disease-related hospitalization during the previous 12 months and the number of such hospitalizations. Because sufficiently complete and harmonized objective clinical indicators were not available in the analytic dataset, the manuscript does not interpret the control indicator as an objectively verified clinical endpoint.

The two proposed intermediate variables were health information acquisition and medical service accessibility. Health information acquisition captured whether patients could obtain timely, understandable, and actionable information about chronic disease management, symptom changes, medication, lifestyle adjustment, follow-up, and referral pathways. Medical service accessibility captured perceived convenience of primary care use, timely consultation, waiting time, follow-up, and referral completion. Both were constructed as multi-item composite scores. Their indirect associations were examined descriptively in an exploratory cross-sectional pathway analysis and were not interpreted as evidence of temporal ordering or a causal indirect pathway.

The main grouping and moderating variables were income, education, and digital literacy. Income was measured using household income categories and recoded into low-, middle-, and high-income groups according to the sample distribution. Education was measured by the highest completed education level and grouped into primary school or below, junior high school, and senior high school or above. Digital literacy was measured using items related to smartphone use, online appointment ability, WeChat use, online health information reading, and internet medical service use. A digital literacy index was created by summing or averaging standardized item scores. The index was divided into low- and high-digital-literacy groups using the median split, with tertile grouping used in robustness checks.

Control variables included age, gender, marital status, living arrangement, residence type, medical insurance type, distance to the primary healthcare institution, chronic disease type, disease duration, number of chronic diseases, baseline health status, and whether the patient lived alone. These covariates were selected on theoretical grounds because they may be associated with both AI service use and health outcomes. Family support and baseline self-management motivation were not directly measured in the questionnaire and, therefore, could not be included as covariates. Their omission is explicitly treated as a potential source of residual unobserved confounding. For newly constructed scales, internal consistency was assessed using Cronbach's alpha and McDonald's omega, and exploratory factor analysis was used to assess dimensionality.

Data cleaning was completed before statistical analysis. The research team first checked each questionnaire for completeness, duplicate entries, out-of-range values, and logical inconsistency. Questionnaires were excluded if they had missing values on core variables, including AI exposure, main outcome variables, income, education, or digital literacy; if they contained duplicate participant codes; or if responses showed a clear logical contradiction, such as reporting no chronic disease while also reporting chronic disease medication and follow-up. For non-core covariates with limited missingness, missing values were handled using complete-case analysis in the main models. If the missing rate of a covariate exceeded 5%, multiple imputation by chained equations was used as a sensitivity analysis under the assumption that data were missing at random. The number and proportion of missing values for all key variables were reported in the Supplementary material.

All quantitative analyses were conducted using Stata version 17. Two-sided tests were used with a significance level of 0.05. The analyses included multivariable regression, subgroup and interaction models, exploratory pathway analysis with non-parametric bootstrap confidence intervals, and pre-specified robustness checks. Qualitative transcripts were organized and coded using a standardized coding matrix and thematic matrices.

### 4.5 Ethical considerations and data quality control

The study protocol was reviewed and approved by the Ethics Committee of Guangdong Medical University before formal data collection. If participating hospitals or primary healthcare institutions required additional local approval, site-level ethical or administrative approval was also obtained. The study followed the ethical principles of the Declaration of Helsinki for medical research involving human participants (27). Because the study involved older adults, chronic disease information, and possible linkage with institutional health records, special attention was paid to informed consent, data minimization, privacy protection, and secure data storage.

All participants received a written information sheet explaining the study purpose, procedures, voluntary nature of participation, potential risks and benefits, confidentiality arrangements, and the right to withdraw at any time without affecting their medical care. Written informed consent was obtained before questionnaire administration or interview recording. For older adults with limited literacy, investigators read the consent form aloud and confirmed understanding before consent was obtained. Participants with severe cognitive impairment who could not provide informed consent were not enrolled. No invasive procedure or clinical intervention was conducted by the research team.

Data confidentiality was strictly maintained. Each participant was assigned a unique study identification code. Names, phone numbers, medical record numbers, and other direct identifiers were not included in the analytic dataset. If objective clinical indicators were extracted from institutional records, a separate linkage file was stored in encrypted form and accessible only to the principal investigator or designated data manager. Interview recordings were transcribed and anonymized. Consent forms, questionnaires, interview transcripts, and clinical abstraction files were stored separately. Only de-identified data were used for statistical analysis.

Several procedures were implemented to ensure data quality. First, all investigators received standardized training before fieldwork, including eligibility screening, informed consent, questionnaire administration, communication with older adults, neutral probing, and handling of missing or inconsistent responses. Second, a fieldwork manual was prepared to ensure consistent procedures across institutions. Third, completed questionnaires were checked on the same day for missing key variables, logical inconsistency, and unusually short completion time. Fourth, 10% of the questionnaires were randomly selected for verification. Fifth, double data entry or automated logic checks were used to reduce data entry errors. Sixth, interview transcripts were checked against audio recordings to ensure accuracy.

To reduce selection bias, recruitment was distributed across weekdays and clinic periods. Standardized wording and neutral probing were used to reduce information and interviewer bias, and participants were informed that identifiable responses would not be shared with their physicians or institutions. Institutional matching, multivariable adjustment, subgroup analysis, alternative exposure definitions, and propensity score weighting were used to improve comparability with respect to observed characteristics. These procedures could not eliminate confounding by unmeasured characteristics. Accordingly, the results are interpreted as adjusted associations and exploratory pathways rather than causal relationships.

## 5 Empirical strategy

### 5.1 Baseline regression model

The empirical strategy examined whether AI-assisted hierarchical diagnosis and treatment were associated with patient-reported chronic disease control, self-rated health, and hospitalization-related outcomes, and whether the associations varied across socioeconomic, digital-literacy, disease-type, and urban-rural groups. The matched comparative observational framework emphasized explicit exposure definition, covariate adjustment, transparent model specification, and cautious interpretation of associations (23).

The baseline model estimates the relationship between AI-assisted hierarchical diagnosis and treatment and patient health outcomes. Let i denote the patient, j denote the healthcare institution, and *p* denote the matched institutional pair. The primary empirical specification is expressed as follows:


$$\begin{array}{l} \text{E}\left({\text{Y}}_{\text{ijp}}\right)=\alpha +\beta {\text{AI}}_{\text{ijp}}+\gamma {\text{X}}_{\text{ijp}}+\mu_{\text{p}}+\varepsilon_{\text{ijp}} \end{array}$$

where Y_ijp_ represents the health outcome of patient i in institution j and matched pair *p*; AI_ijp_ denotes AI exposure or AI use intensity; X_ijp_ is a vector of patient-level covariates; μ_p_ represents matched-pair fixed effects; and ε_ijp_ is the error term. The coefficient beta captures the adjusted association between AI-assisted hierarchical diagnosis and treatment and the outcome of interest. The matched-pair fixed effects are included to account for observable and unobservable characteristics shared within each AI-enabled institution and its matched comparison institution, such as local socioeconomic conditions, geographic context, and general healthcare environment.

Model families were selected according to outcome measurement. Ordinary least squares regression was used for approximately continuous outcomes, including self-rated health and the two composite pathway scores. Logistic regression was used for binary outcomes, including patient-reported disease control and hospitalization. Modified Poisson regression with robust standard errors was used as a complementary specification for binary outcomes (28). Poisson or negative binomial regression was used for hospitalization counts, depending on overdispersion.

The exposure was operationalized in two ways. The primary indicator was institutional AI availability. Secondary analyses used binary individual AI use and an ordinal or standardized AI-use-intensity measure based on self-reported exposure to intelligent guidance, assisted triage, follow-up reminders, risk warning, referral support, and AI-related health consultation. This distinction separates the institutional opportunity to receive AI-supported care from patients' actual engagement with specific services. Because individual use is potentially selective, those estimates are interpreted as supplementary adjusted associations rather than as identified per-protocol relationships.

Baseline models adjusted for age, gender, marital status, living arrangement, residence type, education, income, medical insurance type, chronic disease type, disease duration, number of chronic diseases, baseline health status, distance to the primary healthcare institution, and living alone. Family support and self-management motivation were not measured and could not be adjusted for. Matched-pair fixed effects and robust standard errors were used. Given the small number of institutions, the analysis does not claim that cluster-level or individual-level unobserved confounding was eliminated.

### 5.2 Group regression and interaction analysis

The second part of the empirical strategy examined whether adjusted associations varied across income, education, digital literacy, disease type, and urban-rural residence. These analyses were equity-oriented but exploratory because the number of institutions and some subgroup sample sizes were limited. Larger estimates in disadvantaged groups were interpreted as patterns consistent with a potentially compensatory association, not as proof that AI reduced health inequality.

Two complementary approaches were specified. First, the baseline models were estimated separately by income, education, digital literacy, hypertension status, diabetes status, and urban-rural residence. Disease-specific models treated hypertension, control, and diabetes control separately rather than combining their clinical meanings. Second, interaction terms between institutional AI availability and each grouping variable were used to assess whether subgroup differences were statistically distinguishable. Adjusted estimates, 95% confidence intervals, predicted probabilities or marginal means, and interaction *p*-values were reported where estimable.


$$\begin{array}{l} \text{E}\left({\text{Y}}_{\text{ijp}}\right)=\alpha_{\text{g}}+\beta_{\text{g}}{\text{AI}}_{\text{ijp}}+\gamma_{\text{g}}{\text{X}}_{\text{ijp}}+\mu_{\text{p}}+\varepsilon_{\text{ijp}},\text{for group g} \end{array}$$

Interaction models were used to assess whether the adjusted association between institutional AI availability and each outcome differed across the specified groups. For categorical variables with more than two levels, indicator variables and corresponding interaction terms were included.


$$\begin{array}{l} \text{E}\left({\text{Y}}_{\text{ijp}}\right)=\alpha +\beta_{1}{\text{AI}}_{\text{ijp}}+\beta_{2}{\text{Group}}_{\text{ijp}}+\beta_{3}\left({\text{AI}}_{\text{ijp}}\times {\text{Group}}_{\text{ijp}}\right)\\ \;+\gamma_{\text{ijp}}+\mu_{\text{p}}+\varepsilon_{\text{ijp}} \end{array}$$

In this model, Group_ijp_ represents the subgroup indicator, such as low income, low education, or low digital literacy. The coefficient β_3_ captures whether the association between AI-assisted hierarchical diagnosis and treatment and the outcome differs across the specified subgroup. For categorical subgroup variables with more than two categories, dummy variables and their interactions with AI exposure will be included. Predicted probabilities, adjusted means, or average marginal effects will be reported to improve interpretability, especially for logistic and modified Poisson models.

Health equity-related patterns were evaluated using both interaction tests and the direction and magnitude of adjusted group differences. A smaller disadvantaged-advantaged gap in AI-enabled institutions was described as a pattern consistent with a potentially equity-enhancing association. Because the design was observational, such patterns were not interpreted as causal evidence that AI narrowed disparities.

### 5.3 Exploratory pathway analysis

The third part of the empirical strategy examined whether the observed associations were statistically consistent with proposed indirect pathways through health information acquisition and medical service accessibility. These variables were measured contemporaneously with the exposure and outcomes; they are therefore described as proposed intermediate variables rather than variables establishing a temporal sequence.

The exploratory pathway analysis was conducted in two stages. The first model estimated the adjusted association between AI exposure and each proposed intermediate variable:


$$\begin{array}{l} {\text{M}}_{\text{ijp}}=\alpha_{0}+\alpha_{1}{\text{AI}}_{\text{ijp}}+\gamma {\text{X}}_{\text{ijp}}+\mu_{\text{p}}+\eta_{\text{ijp}} \end{array}$$

where M_ijp_ represents either health information acquisition or medical service accessibility. A positive and statistically significant α_1_ would indicate that AI exposure is associated with improvement in the proposed mechanism variable after covariate adjustment.

The second model estimates the association between AI exposure, the mediator, and the health outcome:


$$\begin{array}{l} \text{E}\left({\text{Y}}_{\text{ijp}}\right)=\beta_{0}+\sigma {\text{AI}}_{\text{ijp}}+\beta_{2}{\text{M}}_{\text{ijp}}+\gamma {\text{X}}_{\text{ijp}}+\mu_{\text{p}}+\varepsilon_{\text{ijp}} \end{array}$$

In this model, β_2_ represents the adjusted association between the proposed intermediate variable and the outcome, while sigma represents the AI-outcome association after including that variable. The product α_1_ × β_2_ was reported as an indirect association, with confidence intervals obtained through non-parametric bootstrap resampling. This quantity is a statistical decomposition of cross-sectional associations and does not establish temporal ordering or a causal pathway.

No causal indirect-pathway estimand was claimed because temporal precedence and sequential ignorability could not be established. In particular, unmeasured characteristics may confound the exposure-intermediate-variable and intermediate-variable-outcome associations. The analysis, therefore, uses the terms total association, association adjusted for the proposed pathway variable, and indirect association. Health information acquisition and medical service accessibility were first examined separately and then jointly as proposed intermediate variables. Correlations and multicollinearity diagnostics were assessed before interpreting the joint specification. Pathway analyses were also considered across digital-literacy groups to assess whether the observed indirect associations varied with patients' digital capabilities.

### 5.4 Robustness checks

A series of robustness checks will be conducted to assess the stability of the empirical findings. First, the exposure variable will be redefined in alternative ways. The main analysis will use institutional AI access and patient-level AI use intensity. Robustness analyses will separately use a binary indicator of any AI use, an ordinal frequency measure, and a standardized AI-use index. Patients who report that they are “not sure” whether they have used AI-assisted services will be excluded in one sensitivity analysis and recoded as non-users in another to examine whether classification uncertainty affects the results.

Second, alternative outcome specifications will be used. For self-rated health, both continuous linear regression and ordinal logistic regression will be estimated. For chronic disease control and hospitalization, logistic regression and modified Poisson regression will be compared. For hospitalization counts, Poisson and negative binomial models will be estimated, and the latter will be preferred if overdispersion is detected. If objective clinical indicators such as blood pressure readings, fasting blood glucose, or HbA1c are available for part of the sample, the analysis will be repeated using these indicators as robustness outcomes.

Third, confounding will be addressed through additional adjustment and propensity score methods. Because patients in AI-enabled and comparison institutions may differ in baseline characteristics, propensity scores will be estimated using sociodemographic characteristics, health status, chronic disease profile, insurance type, distance to primary healthcare institutions, and baseline service-use indicators. Propensity score matching, inverse probability of treatment weighting, or covariate balancing will be used as sensitivity analyses. Covariate balance before and after adjustment will be assessed using standardized mean differences, with values below 0.10 generally interpreted as acceptable balance (29).

Fourth, missing data will be examined carefully. The amount and pattern of missingness will be reported for all key variables. If missingness is low, complete-case analysis will be used as the main approach. If missingness is non-negligible and plausibly missing at random, multiple imputation will be used as a robustness check, following the logic that multiple imputation can reduce bias and improve efficiency compared with complete-case analysis when its assumptions are reasonable (30). Results from the complete-case and imputed datasets will be compared.

Fifth, model diagnostics will be performed. Multicollinearity will be assessed using variance inflation factors. Heteroskedasticity-robust standard errors will be used for linear models, following the logic of robust covariance estimation (31). Influential observations will be identified using leverage and residual diagnostics. For composite scales, internal consistency will be assessed using Cronbach's alpha and McDonald's omega, since omega is often recommended as a more flexible reliability estimate when tau-equivalence assumptions are not fully satisfied (32).

Finally, the heterogeneity results will be examined using alternative group definitions. Income groups will be redefined using tertiles instead of pre-defined categories. Digital literacy will be analyzed using both median split and tertile grouping. Education will be treated both as a categorical variable and as an ordered variable. If the substantive conclusions remain consistent across these specifications, the findings will be considered more robust.

### 5.5 Qualitative interview coding and triangulation

The qualitative component contextualized the quantitative findings and examined implementation differences across AI functions, professional roles, and institutional workflows. Reporting followed the COREQ framework, including respondent selection, institutional context, data collection, coding, saturation assessment, and integration with quantitative findings (22). All interviews were audio-recorded with consent, transcribed verbatim, checked against the recordings, and anonymized. A hybrid thematic-analysis strategy combined deductive codes from the conceptual framework with inductive codes emerging from the transcripts. The deductive codebook distinguished intelligent guidance, assisted triage, referral support, follow-up reminders, risk warning, clinical decision support, digital-literacy barriers, provider workload, and equity concerns.

Two trained researchers independently coded an initial subset of transcripts and conducted code-by-code comparisons. Discrepancies were documented, discussed, and resolved by consensus, after which the code definitions and decision rules were revised (33). The remaining transcripts were coded with the stabilized codebook, and ambiguous passages were reviewed jointly. Because the analysis used a consensus-oriented thematic procedure rather than a fixed-category reliability design, no formal kappa coefficient was calculated; this is now reported transparently. Thematic saturation was assessed iteratively by tracking the appearance of new codes and major themes. Later interviews elaborated role- and function-specific variation within the three established themes but did not require an additional major theme.

Triangulation was conducted across respondent roles, institutions, AI functions, and methods. Quantitative associations were compared with interview evidence to determine whether the qualitative material converged with, complemented, expanded, or contradicted the survey findings (24). Function-specific interpretation distinguished patient-facing guidance and triage from longitudinal follow-up, risk-warning, referral-support, and clinician-facing decision-support functions.

Integration was implemented through joint displays linking each quantitative finding with relevant qualitative themes and AI functions. Convergence indicated a similar pattern across methods; complementarity provided additional context; expansion identified implementation conditions not captured by the questionnaire; and discrepancy identified patterns requiring cautious interpretation. The qualitative findings were not treated as statistical or causal evidence. They were used to contextualize how institutional workflows, staff-assisted implementation, and particular AI functions could be related to the quantitative patterns observed in the survey data.

## 6 Results

### 6.1 Descriptive statistics

Table 1 presents the baseline characteristics of the analytic sample by institutional AI exposure status. The final sample included 420 older adults with chronic diseases, with 210 participants recruited from AI-enabled primary healthcare institutions and 210 from matched comparison institutions. The mean age of the respondents was 69.8 years (SD = 6.4), and 54.5% were female. Most participants were married (74.3%) and did not live alone (81.4%). In terms of educational attainment, 42.4% had primary school education or below, 35.0% had junior high school education, 15.0% had senior high school or technical school education, and 7.6% had college education or above. Regarding household income, 35.0% of participants were classified as low income, 39.5% as middle income, and 25.5% as high income. Slightly less than half of the sample had low digital literacy (48.6%). Hypertension was the most frequently reported chronic condition (66.7%), followed by diabetes (44.8%), cardiovascular disease (23.6%), chronic respiratory disease (14.5%), stroke sequelae (11.4%), and chronic kidney disease (8.3%). Because chronic disease categories were not mutually exclusive, these percentages do not add up to 100%. Multimorbidity was reported by 44.3% of participants.

**Table 1:** Baseline characteristics of participants by institutional AI exposure status.

Variable	Full sample (*n* = 420)	AI-enabled group (*n* = 210)	Comparison group (*n* = 210)	*p*-value
Age, years				0.337
Mean (SD)	69.8 (6.4)	69.5 (6.2)	70.1 (6.6)	
Sex, *n* (%)				0.769
Male	191 (45.5)	94 (44.8)	97 (46.2)	
Female	229 (54.5)	116 (55.2)	113 (53.8)	
Marital status, *n* (%)				0.503
Married	312 (74.3)	159 (75.7)	153 (72.9)	
Not married	108 (25.7)	51 (24.3)	57 (27.1)	
Living arrangement, *n* (%)				0.616
Living alone	78 (18.6)	37 (17.6)	41 (19.5)	
Not living alone	342 (81.4)	173 (82.4)	169 (80.5)	
Education level, *n* (%)				0.943
Primary school or below	178 (42.4)	86 (41.0)	92 (43.8)	
Junior high school	147 (35.0)	76 (36.2)	71 (33.8)	
Senior high school/technical school	63 (15.0)	32 (15.2)	31 (14.8)	
College or above	32 (7.6)	16 (7.6)	16 (7.6)	
Household income level, *n* (%)				0.954
Low income	147 (35.0)	72 (34.3)	75 (35.7)	
Middle income	166 (39.5)	84 (40.0)	82 (39.0)	
High income	107 (25.5)	54 (25.7)	53 (25.2)	
Digital literacy level, *n* (%)				0.558
Low digital literacy	204 (48.6)	99 (47.1)	105 (50.0)	
High digital literacy	216 (51.4)	111 (52.9)	105 (50.0)	
Chronic disease profile, *n* (%)				
Hypertension	280 (66.7)	142 (67.6)	138 (65.7)	0.679
Diabetes	188 (44.8)	93 (44.3)	95 (45.2)	0.844
Cardiovascular disease	99 (23.6)	48 (22.9)	51 (24.3)	0.73
Chronic respiratory disease	61 (14.5)	30 (14.3)	31 (14.8)	0.89
Stroke sequelae	48 (11.4)	23 (11.0)	25 (11.9)	0.759
Chronic kidney disease	35 (8.3)	16 (7.6)	19 (9.0)	0.596
Multimorbidity, *n* (%)				0.556
No	234 (55.7)	120 (57.1)	114 (54.3)	
Yes	186 (44.3)	90 (42.9)	96 (45.7)	
AI service use, *n* (%)				< 0.001
No AI service use	254 (60.5)	44 (21.0)	210 (100.0)	
Any AI service use	166 (39.5)	166 (79.0)	0 (0.0)	
Chronic disease control, *n* (%)				0.008
Not controlled	144 (34.3)	59 (28.1)	85 (40.5)	
Controlled	276 (65.7)	151 (71.9)	125 (59.5)	
Self-rated health				0.002
Mean (SD)	3.31 (0.91)	3.45 (0.88)	3.17 (0.92)	
Hospitalization in past 12 months, *n* (%)				0.073
No	331 (78.8)	173 (82.4)	158 (75.2)	
Yes	89 (21.2)	37 (17.6)	52 (24.8)	
Health information acquisition				< 0.001
Mean (SD)	3.58 (0.73)	3.83 (0.68)	3.33 (0.70)	
Medical service accessibility				< 0.001
Mean (SD)	3.49 (0.76)	3.74 (0.71)	3.25 (0.74)	

The AI-enabled and comparison groups were broadly comparable in major sociodemographic and disease-profile characteristics. No statistically significant between-group differences were observed in age, sex, marital status, living arrangement, education level, household income, digital literacy, individual chronic disease categories, or multimorbidity. This pattern suggests that the matched institutional sampling strategy achieved a reasonable baseline balance between the two groups, although residual confounding cannot be fully ruled out in a non-randomized observational design. As expected, AI service exposure differed substantially between groups: 79.0% of participants in AI-enabled institutions reported using at least one AI-related service, whereas no participants in the comparison group reported AI service use (*p* < 0.001). In terms of outcome-related variables, the AI-enabled group showed a higher chronic disease control rate than the comparison group (71.9% vs. 59.5%, *p* = 0.008) and a higher mean self-rated health score (3.45 vs. 3.17, *p* = 0.002). The proportion of participants reporting chronic disease-related hospitalization in the past 12 months was lower in the AI-enabled group than in the comparison group (17.6% vs. 24.8%), although this difference did not reach the conventional 0.05 level of statistical significance (*p* = 0.073). Patients in AI-enabled institutions also reported higher health information acquisition scores (3.83 vs. 3.33, *p* < 0.001) and higher medical service accessibility scores (3.74 vs. 3.25, *p* < 0.001), providing preliminary descriptive evidence for the proposed information and accessibility mechanisms.

### 6.2 Baseline associations of AI-assisted hierarchical diagnosis and treatment

Table 2 reports the adjusted associations between AI-assisted hierarchical diagnosis and treatment and health outcomes. After adjustment for sociodemographic characteristics, chronic disease profile, insurance type, baseline health status, distance to primary healthcare institutions, and matched-pair fixed effects, institutional AI access was positively associated with chronic disease control. The adjusted odds ratio was 1.69 (95% CI: 1.12–2.56, *p* = 0.013), suggesting that patients from AI-enabled institutions were more likely to report controlled blood pressure or blood glucose than patients from comparison institutions.

**Table 2:** Baseline associations between AI-assisted hierarchical diagnosis and treatment and health outcomes.

Variables	Chronic disease control OR (95% CI)	Self-rated health β (95% CI)	Hospitalization OR (95% CI)	Number of hospitalizations IRR (95% CI)
Institutional AI access	1.69^*^ (1.12 to 2.56)	0.24^**^ (0.08 to 0.39)	0.63^*^ (0.39 to 0.99)	0.70^*^ (0.50 to 0.99)
Age	0.98 (0.95 to 1.01)	−0.01 (−0.02 to 0.00)	1.03^*^ (1.00 to 1.06)	1.02 (0.99 to 1.05)
Female	1.08 (0.73 to 1.60)	0.03 (−0.11 to 0.17)	0.94 (0.59 to 1.51)	0.97 (0.70 to 1.35)
Junior high school	1.23 (0.78 to 1.93)	0.10 (−0.07 to 0.27)	0.86 (0.50 to 1.48)	0.90 (0.61 to 1.32)
Senior high school or above	1.41 (0.83 to 2.41)	0.18^*^ (0.01 to 0.35)	0.78 (0.42 to 1.46)	0.83 (0.53 to 1.30)
Middle income	1.18 (0.75 to 1.86)	0.09 (−0.08 to 0.25)	0.91 (0.54 to 1.54)	0.95 (0.65 to 1.38)
High income	1.32 (0.77 to 2.26)	0.16 (0.00 to 0.33)	0.80 (0.42 to 1.51)	0.87 (0.55 to 1.36)
High digital literacy	1.36^*^ (1.01 to 1.84)	0.20^**^ (0.07 to 0.33)	0.82 (0.55 to 1.22)	0.86 (0.63 to 1.17)
Multimorbidity	0.73 (0.50 to 1.08)	−0.16^*^ (−0.31 to −0.01)	1.64^**^ (1.03 to 2.62)	1.55^**^ (1.12 to 2.16)
Distance to primary institution	0.94 (0.87 to 1.02)	−0.03^*^ (−0.06 to −0.01)	1.06 (0.97 to 1.16)	1.07 (0.99 to 1.15)
Matched-pair fixed effects	Yes	Yes	Yes	Yes
Observations	420	420	420	420

OR, odds ratio; IRR, incidence rate ratio; CI, confidence interval. The reference categories are primary school or below, low income, and low digital literacy. Models adjusted for age, gender, marital status, living arrangement, residence type, insurance type, chronic disease type, disease duration, baseline health status, living alone, and matched-pair fixed effects. ^*^p < 0.05, ^**^p < 0.01.

AI access was also positively associated with self-rated health. The adjusted coefficient was 0.24 (95% CI: 0.08–0.39, *p* = 0.003), representing an adjusted difference in self-rated health scores rather than an identified causal change. Institutional AI access was associated with lower odds of chronic disease-related hospitalization (OR = 0.63, 95% CI: 0.39–0.99, *p* = 0.047), and the hospitalization-count model produced a directionally similar association (IRR = 0.70, 95% CI: 0.50–0.99, *p* = 0.044). These findings were consistent with H1.

### 6.3 Health equity-related associations across income, education, and digital literacy groups

Table 3 presents subgroup analyses across income, education, and digital-literacy groups. The adjusted association with patient-reported chronic disease control was larger in the low-income group (OR = 2.02, 95% CI: 1.15–3.57) than in the high-income group (OR = 1.32, 95% CI: 0.70–2.50). This pattern is consistent with the possibility that AI-supported primary care is particularly relevant to economically disadvantaged patients, but it does not establish that AI caused a reduction in income-related disparities.

**Table 3:** Heterogeneous effects of AI-assisted hierarchical diagnosis and treatment.

Subgroup	Chronic disease control OR (95% CI)	Self-rated health β (95% CI)	Hospitalization OR (95% CI)	Interaction *p*-value
Low income	2.02^*^ (1.15 to 3.57)	0.31^*^ (0.07 to 0.55)	0.55^*^ (0.31 to 0.98)	0.041
Middle income	1.60^*^ (1.01 to 2.54)	0.22^*^ (0.02 to 0.42)	0.67 (0.38 to 1.18)	—
High income	1.32 (0.70 to 2.50)	0.16 (−0.07 to 0.39)	0.79 (0.38 to 1.63)	—
Primary school or below	1.96^*^ (1.12 to 3.45)	0.29^*^ (0.05 to 0.53)	0.58^*^ (0.34 to 0.99)	0.048
Junior high school	1.53 (0.91 to 2.58)	0.21 (0.00 to 0.42)	0.69 (0.39 to 1.22)	—
Senior high school or above	1.29 (0.67 to 2.49)	0.14 (−0.09 to 0.37)	0.83 (0.40 to 1.71)	—
Low digital literacy	1.54 (0.95 to 2.49)	0.17 (−0.02 to 0.36)	0.72 (0.43 to 1.21)	0.216
High digital literacy	1.84^*^ (1.10 to 3.08)	0.30^**^ (0.11 to 0.49)	0.56^*^ (0.32 to 0.98)	—

Each row reports the adjusted association between institutional AI access and the corresponding outcome within the subgroup. Interaction p-values refer to the interaction between AI access and the disadvantaged subgroup indicator. ^*^p < 0.05, ^**^p < 0.01.

A similar pattern was observed in education. Among patients with primary school education or below, AI access was associated with higher odds of chronic disease control (OR = 1.96, 95% CI: 1.12–3.45), whereas the estimate was smaller among those with senior high school education or above (OR = 1.29, 95% CI: 0.67–2.49). These results suggest that AI-assisted guidance and follow-up may help reduce information and navigation barriers among patients with lower educational attainment.

Digital literacy showed a somewhat different pattern. AI access was beneficial in both low- and high-digital-literacy groups, but the estimated association was slightly stronger among patients with high digital literacy. The adjusted odds ratio was 1.54 (95% CI: 0.95–2.49) among low-digital-literacy patients and 1.84 (95% CI: 1.10–3.08) among high-digital-literacy patients. This finding suggests that AI-enabled services may improve average health outcomes, but digital capability may still condition the extent to which patients benefit from these services.

The adjusted predicted probabilities shown in Figure 2 further illustrate the equity pattern. The predicted probability of chronic disease control was higher in the AI-enabled group across all subgroups. The absolute difference was especially visible among low-income patients and patients with primary school education or below. For digital literacy, the AI-enabled group also showed higher predicted probabilities in both low- and high-literacy groups, although the gap remained larger among high-digital-literacy patients. This result points to a cautious interpretation: AI-enabled hierarchical diagnosis and treatment may help reduce income- and education-related gaps, but digital exclusion remains a potential boundary condition.

**Figure 2:**
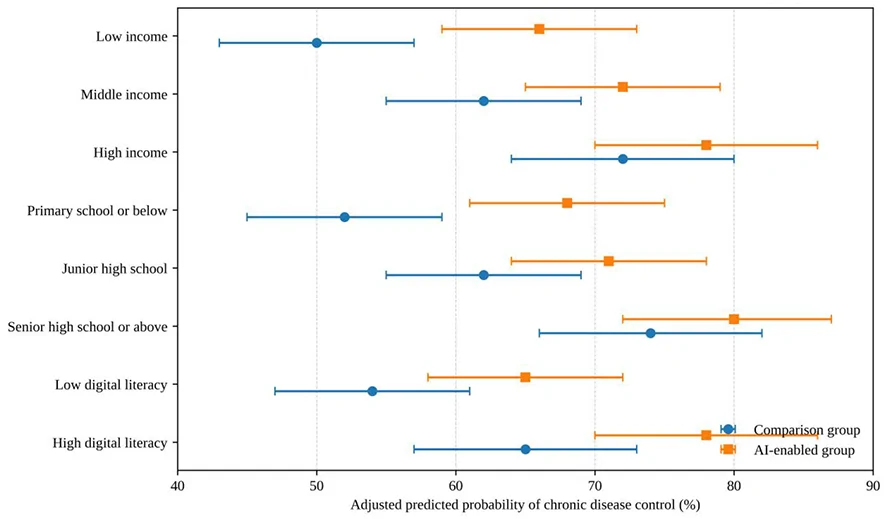
Adjusted predicted probability of chronic disease control by subgroup.

Additional subgroup analyses were conducted to examine whether the association between institutional AI access and patient-reported chronic disease control varied by disease type and place of residence. Disease-specific models were estimated separately for participants with hypertension and diabetes. For participants with hypertension, the outcome was defined as self-reported blood pressure control, whereas for participants with diabetes, the outcome was defined as self-reported blood glucose control. Participants with both conditions were included in each applicable disease-specific model, with the corresponding control outcome evaluated separately. Because the disease categories were not mutually exclusive and the clinical definitions of disease control differed between hypertension and diabetes, no direct interaction test comparing the two disease groups was performed. As shown in Table 4, institutional AI access was associated with higher odds of self-reported blood pressure control among participants with hypertension (adjusted OR = 1.76, 95% CI: 1.05–2.96, *p* = 0.033). Among participants with diabetes, the estimated association with self-reported blood glucose control was also positive, although the confidence interval included the null value (adjusted OR = 1.63, 95% CI: 0.87–3.05, *p* = 0.126). The directionally similar estimates suggest that the overall association with chronic disease control was not attributable exclusively to either hypertension or diabetes. However, the wider confidence interval in the diabetes subgroup indicates lower statistical precision, partly because of its smaller sample size. The residence-stratified analyses also showed positive associations in both urban and rural settings. Institutional AI access was associated with higher odds of patient-reported chronic disease control among urban residents (adjusted OR = 1.51, 95% CI: 0.92–2.48, *p* = 0.103) and rural residents (adjusted OR = 2.02, 95% CI: 1.07–3.81, *p* = 0.030). Although the point estimate was larger among rural residents, the interaction between institutional AI access and residence type was not statistically significant (*p* for interaction = 0.480). Therefore, the results do not provide sufficient evidence that the association differed between urban and rural residents. The comparatively larger point estimate in the rural subgroup should be interpreted as exploratory rather than as evidence of a differential effect.

**Table 4:** Disease-specific and urban–rural subgroup analyses of patient-reported chronic disease control.

Analysis	Subgroup	Full sample, *n*	AI-enabled, *n*	Comparison, *n*	Adjusted OR (95% CI)	*p*-value	*p* for interaction
Disease-specific analysis	Hypertension	280	142	138	1.76 (1.05 to 2.96)	0.033	—
	Diabetes	188	93	95	1.63 (0.87 to 3.05)	0.126	—
Residence-stratified analysis	Urban residence	252	128	124	1.51 (0.92 to 2.48)	0.103	0.48
	Rural residence	168	82	86	2.02 (1.07 to 3.81)	0.03	—

OR, odds ratio; CI, confidence interval. Disease-specific models used self-reported blood pressure control as the outcome for participants with hypertension and self-reported blood glucose control as the outcome for participants with diabetes. Because chronic disease categories were not mutually exclusive, the disease-specific sample sizes do not sum to the full analytic sample.

### 6.4 Exploratory pathway analysis: health information acquisition

Table 5 reports the exploratory cross-sectional pathway analysis involving health information acquisition. Institutional AI access was positively associated with health information acquisition (beta = 0.43, 95% CI: 0.31–0.55, *p* < 0.001). This association was consistent with H2a and indicated that participants in AI-enabled institutions reported better access to timely, understandable, and actionable chronic disease information.

**Table 5:** Exploratory pathway analysis through health information acquisition.

Outcome	Total association	Association adjusted for pathway variable	Indirect association	Bootstrap 95% CI	Proportion of total association
Chronic disease control	0.525^*^	0.394	0.131^*^	0.046 to 0.244	25.00%
Self-rated health	0.240^**^	0.173^*^	0.067^*^	0.026 to 0.121	27.90%
Hospitalization	−0.462^*^	−0.352	−0.110^*^	−0.210 to −0.032	23.80%

Effects for chronic disease control and hospitalization are reported on the log-odds scale. Effects for self-rated health are reported as linear coefficients. Indirect effects were estimated using non-parametric bootstrap confidence intervals. ^*^p < 0.05, ^**^p < 0.01.

Health information acquisition was also associated with the health outcomes after covariate adjustment. The bootstrapped indirect association was 0.131 (95% CI: 0.046–0.244) for patient-reported chronic disease control, 0.067 (95% CI: 0.026–0.121) for self-rated health, and −0.110 (95% CI: −0.210 to −0.032) for hospitalization. These cross-sectional estimates were statistically consistent with the pathway proposed in H2b.

### 6.5 Exploratory pathway analysis: medical service accessibility

Table 6 reports the exploratory cross-sectional pathway analysis involving medical service accessibility. Institutional AI access was positively associated with perceived medical service accessibility (beta = 0.39, 95% CI: 0.27–0.51, *p* < 0.001), a pattern consistent with H3a.

**Table 6:** Exploratory pathway analysis through medical service accessibility.

Outcome	Total association	Association adjusted for pathway variable	Indirect association	Bootstrap 95% CI	Proportion of total association
Chronic disease control	0.525^*^	0.421^*^	0.104^*^	0.030 to 0.206	19.80%
Self-rated health	0.240^**^	0.179^*^	0.061^*^	0.019 to 0.113	25.40%
Hospitalization	−0.462^*^	−0.368	−0.094^*^	−0.181 to −0.018	20.30%

Effects for chronic disease control and hospitalization are reported on the log-odds scale. Effects for self-rated health are reported as linear coefficients. Indirect effects were estimated using non-parametric bootstrap confidence intervals. ^*^p < 0.05, ^**^p < 0.01.

Medical service accessibility was also associated with the outcomes after covariate adjustment. The bootstrapped indirect association was 0.104 (95% CI: 0.030–0.206) for patient-reported chronic disease control, 0.061 (95% CI: 0.019–0.113) for self-rated health, and −0.094 (95% CI: −0.181 to −0.018) for hospitalization. These estimates were statistically consistent with the pathway proposed in H3b but do not establish temporal ordering or a causal pathway between AI availability, accessibility, and the outcomes.

Figure 3 summarizes the estimated indirect associations involving the two proposed intermediate variables. The confidence intervals did not cross zero, indicating cross-sectional statistical patterns consistent with the proposed pathways. This figure does not establish temporal ordering or a causal pathway.

**Figure 3:**
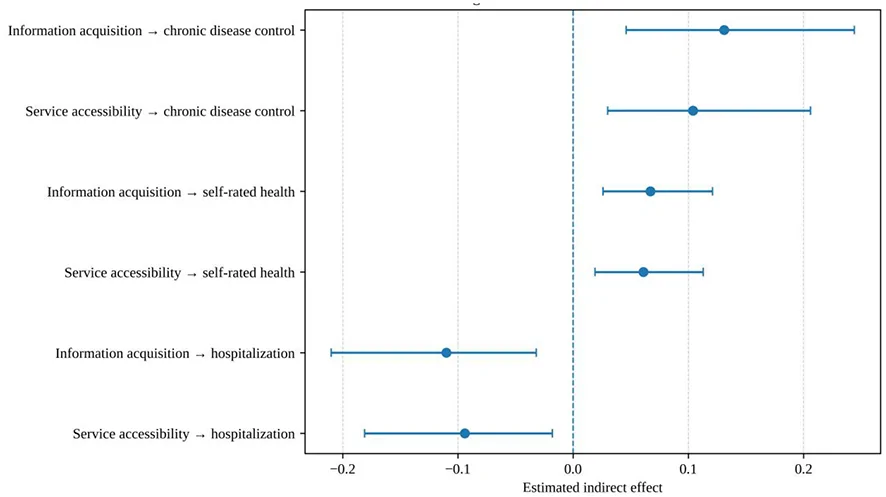
Forest plot of indirect associations through the proposed pathway variables. Points represent estimated indirect effects, and horizontal lines represent 95% bootstrap confidence intervals. Positive values indicate improved chronic disease control or self-rated health, whereas negative values indicate reduced hospitalization risk.

### 6.6 Robustness checks

Table 7 summarizes the robustness checks. First, patient-level AI use intensity was used as an alternative exposure measure. The results remained consistent with the baseline analysis. Higher AI use intensity was associated with higher chronic disease control, better self-rated health, and lower hospitalization risk. Second, binary patient-level AI use produced similar results, suggesting that the findings were not solely driven by institutional classification. Third, alternative model specifications were estimated. Modified Poisson regression produced a risk ratio of 1.17 for chronic disease control and 0.72 for hospitalization, both consistent with the direction of the baseline logistic models. Ordinal logistic regression for self-rated health also produced a positive association. Fourth, propensity score weighting improved covariate balance between AI-enabled and comparison groups, and the estimated associations remained stable. Fifth, multiple imputation for missing values generated results similar to the complete-case analysis. Finally, the heterogeneity patterns remained generally consistent when income and digital literacy were reclassified using tertiles.

**Table 7:** Robustness checks.

Robustness test	Chronic disease control	Self-rated health	Hospitalization	Conclusion
Patient-level AI use intensity	OR = 1.25^*^ (1.08 to 1.45)	β = 0.10^**^ (0.03 to 0.16)	OR = 0.83^*^ (0.70 to 0.98)	Consistent
Binary patient-level AI use	OR = 1.58^*^ (1.06 to 2.36)	β = 0.21^**^ (0.06 to 0.36)	OR = 0.66 (0.42 to 1.03)	Consistent
Modified Poisson model	RR = 1.17^*^ (1.04 to 1.31)	—	RR = 0.72^*^ (0.53 to 0.98)	Consistent
Ordinal logistic model	—	OR = 1.45^**^ (1.11 to 1.89)	—	Consistent
Propensity score weighting	OR = 1.61^*^ (1.05 to 2.48)	β = 0.22^**^ (0.06 to 0.38)	OR = 0.65^*^ (0.41 to 0.99)	Consistent
Multiple imputation	OR = 1.66^*^ (1.10 to 2.50)	β = 0.23^**^ (0.07 to 0.38)	OR = 0.64^*^ (0.40 to 0.99)	Consistent
Alternative subgroup definitions	Similar direction	Similar direction	Similar direction	Partially consistent

OR, odds ratio; RR, risk ratio; CI, confidence interval. ^*^p < 0.05, ^**^p < 0.01.

**Table 8:** Composition and analytic coverage of the qualitative sample.

Characteristic	AI-enabled institutions	Comparison institutions	Total
Institutions	4	4	8
Interviewees	24	24	48
Interviewees per institution	6	6	6
Roles represented	Clinical, chronic disease management, referral, AI operation, and administration	Clinical, chronic disease management, referral, and administration	Multidisciplinary frontline and managerial respondents
Coding approach	Initial independent double-coding followed by consensus codebook	Initial independent double-coding followed by consensus codebook	Consensus-oriented thematic analysis

**Table 9:** AI functions are distinguished in the qualitative analysis.

AI function	Primary target	Analytical distinction
Intelligent guidance	Patients and staff	Care navigation and understandable service guidance
Assisted triage	Patients and triage staff	Initial service matching and prioritization
Follow-up reminders	Patients and chronic disease teams	Continuity and scheduled disease management
Risk warning	Clinical and public health staff	Identification of records requiring review
Referral support	Patients, clinicians, and coordinators	Connection between recommendations and actual referral channels
Clinical decision support	Primary care clinicians	Provider-facing support; not autonomous diagnosis

Overall, the robustness checks provided supportive evidence for the stability of the adjusted associations across alternative exposure definitions, model specifications, observed-confounding adjustments, missing-data approaches, and subgroup classifications. Individual AI use and use intensity were analyzed separately from institutional availability, because individual uptake may be selective; those estimates do not eliminate confounding by self-management motivation, family support, or health-seeking behavior.

### 6.7 Qualitative findings and triangulation

Semi-structured interviews with 48 medical staff members and institutional managers were analyzed using the consensus-oriented thematic procedure described above. The sample included 24 respondents from AI-enabled institutions and 24 from comparison institutions, with six respondents per institution and representation from frontline clinical, chronic-disease-management, referral, AI-operation, and administrative roles. Later interviews expanded role- and function-specific detail but did not require an additional major theme. Three themes were identified: AI-supported routine follow-up and patient guidance; low digital literacy required staff-mediated use; and institutional workflow determined whether AI recommendations were actionable.

#### 6.7.1 Theme 1: AI improved routine follow-up and patient guidance

Medical staff commonly reported that AI-assisted tools helped standardize routine follow-up and provide clearer guidance for older chronic disease patients. In particular, AI-supported reminders, risk alerts, and triage prompts were described as useful for identifying patients who needed timely follow-up or further consultation. This finding helps explain why patients in AI-enabled institutions reported higher health information acquisition and better medical service accessibility. Rather than replacing clinical judgment, AI appeared to function as a workflow support tool that helped staff identify patient needs more quickly and communicate care pathways more clearly.

“The system reminds us which patients need follow-up and which patients may have abnormal blood pressure or blood glucose records. It does not make the decision for us, but it helps us notice problems earlier.” *(GP03, AI-enabled institution)*

“For many older adult patients, the most important thing is not complicated medical knowledge. They want to know whether they should come to the community health center, go to a higher-level hospital, or just continue monitoring. The AI guidance makes this explanation easier.” *(Nurse07, AI-enabled institution)*

This qualitative evidence complemented the exploratory pathway analysis by showing how follow-up reminders, risk alerts, triage prompts, and staff explanation were related to information delivery and routine follow-up. It does not establish that these functions caused the observed health outcomes.

#### 6.7.2 Theme 2: low digital literacy requires staff-mediated AI use

Interviewees also emphasized that many older patients, especially those with lower education or weaker digital skills, could not independently use AI-enabled services. Some patients did not know how to access online guidance, read digital reminders, or complete digital registration. In these cases, AI use depended heavily on assistance from nurses, family doctor teams, community health workers, or family members. This finding is consistent with the quantitative result that digital literacy remained an important boundary condition: AI-enabled services were beneficial, but the benefits were more stable among patients with higher digital literacy.

“Some older patients do not really use the AI system by themselves. They bring their phone, and we help them check the reminder or explain the result. Without this support, they may not know what the message means.” *(Nurse12, AI-enabled institution)*

“For patients who can use smartphones, the system is convenient. For those who cannot, the technology still has to go through us. So the key is not only whether the system exists, but whether someone can help the patient use it.” *(Manager04, AI-enabled institution)*

This theme provides an important qualification to the health equity interpretation. AI-assisted primary care may reduce some income- and education-related barriers when staff use AI outputs to provide clearer explanations and follow-up support. However, if AI services are delivered only through digital interfaces, older adults with low digital literacy may still face exclusion. Therefore, human-supported AI use appears to be essential for making AI-enabled care accessible to digitally disadvantaged patients.

#### 6.7.3 Theme 3: institutional workflow determined whether AI recommendations were actionable

A third theme concerned institutional workflow. Staff members noted that AI recommendations were useful only when they were connected to actual service capacity, referral channels, and follow-up responsibilities. In institutions where AI alerts were integrated into chronic disease management teams, staff could respond to risk signals and adjust follow-up plans. In contrast, if AI recommendations were not clearly assigned to specific staff members or not connected with referral procedures, they were less likely to change patient care. This helps explain why institutional implementation quality may affect the observed association between AI-enabled care and patient outcomes.

“The system can generate a risk alert, but someone must be responsible for checking it. If the alert is not linked to our follow-up work, it may just stay in the system.”*(GP09, AI-enabled institution)*

“AI is helpful when it is connected with our family doctor team and referral process. If the patient needs referral, we still need a real channel and staff coordination. The system alone cannot solve that.”*(Manager08, AI-enabled institution)*

This theme indicates that AI-assisted hierarchical diagnosis and treatment should be understood as a sociotechnical arrangement embedded in institutional routines. The observed associations depend not only on algorithmic output but also on workflows, trained staff, referral coordination, and accountability. AI access was associated with better outcomes, but the pattern likely reflected the combined contribution of technology, staff support, organizational integration, and patient selection.

Overall, the qualitative findings were largely consistent with the quantitative patterns. The first theme complemented the health-information pathway analysis, the second contextualized digital-literacy heterogeneity, and the third showed that associations differed across functional and organizational contexts. Follow-up reminders and risk warnings were primarily linked to longitudinal management, guidance, and triage to patient navigation, and referral and decision support to organizational workflow and professional responsibility (Table 10).

**Table 10:** Joint display of quantitative findings and qualitative themes.

Quantitative finding	Qualitative theme	Interpretation
AI-enabled care was associated with higher health information acquisition scores.	AI improved routine follow-up and patient guidance.	AI may reduce information asymmetry by helping staff provide clearer guidance, reminders, and risk communication.
AI-enabled care was associated with higher medical service accessibility scores.	Institutional workflow determined whether AI recommendations were actionable.	AI may improve accessibility only when recommendations are connected to follow-up teams, referral channels, and service coordination.
AI-related associations appeared less consistent among low-digital-literacy patients.	Low digital literacy required staff-mediated AI use.	Digital literacy remains a boundary condition; human assistance is needed to prevent AI-enabled services from excluding disadvantaged older adults.
AI-enabled care was associated with better chronic disease control and lower hospitalization risk.	AI improved follow-up, but did not replace clinical judgment.	Improved outcomes may reflect better coordination and continuity of care rather than autonomous AI decision-making.

These triangulated findings suggest that AI-assisted hierarchical diagnosis and treatment may contribute to health equity when it is embedded in primary care workflows and supported by medical staff. However, the interviews also indicate that AI implementation is not automatically inclusive. For older chronic disease patients with low digital literacy, the practical benefits of AI depend on staff explanation, simplified access, offline support, and reliable referral coordination.

## 7 Discussion

This study examined associations between AI-enabled primary care, chronic disease management outcomes, and health equity-related differences among older adults. Institutional AI availability was associated with patient-reported chronic disease control, self-rated health, and hospitalization-related outcomes, but the matched observational design does not establish that AI caused these differences. Associations appeared stronger in some low-income and low-education groups, while digital literacy remained an important boundary condition. The exploratory pathway analysis produced cross-sectional indirect associations involving health information acquisition and medical service accessibility; these patterns are hypothesis-generating and do not establish temporal ordering or a causal pathway.

### 7.1 AI-enabled hierarchical diagnosis and treatment, and the coordinating function of primary care

The first finding was an adjusted association between institutional AI availability and the measured outcomes. Institutional AI availability was positively associated with patient-reported chronic disease control and self-rated health and negatively associated with hospitalization. These patterns were generally stable across the reported robustness checks. They should be interpreted as associations that may reflect technology, staff support, organizational capacity, and patient selection rather than as the isolated causal contribution of AI.

This interpretation is consistent with the broader literature on AI in primary care. Recent reviews have suggested that AI can support diagnosis, workflow efficiency, clinical decision-making, chronic disease management, and patient engagement, but its value depends heavily on whether it is embedded into clinical workflow and relational care rather than used as a detached technical product (3). In the present study, the AI systems of interest were not limited to diagnostic algorithms. They were embedded in intelligent guidance, triage, referral support, follow-up reminders, and chronic disease risk warning. These functions correspond closely to the practical difficulties faced by older chronic disease patients: knowing where to seek care, when to follow up, how to interpret symptoms, and whether referral is necessary.

The findings also extend research on China's hierarchical diagnosis and treatment system. Previous studies have reported variation in primary care preferences and perceived quality across patient groups and local contexts (1, 2). Against this background, the present associations are consistent with AI functioning as supportive infrastructure when guidance, follow-up, and service matching are integrated into routine primary care. They should not be interpreted as evidence that AI alone resolves structural weaknesses in professional capacity, referral channels, continuity, or patient trust.

### 7.2 Health equity-related associations are conditional rather than automatic

The second major finding concerned heterogeneity in the adjusted associations. Associations were larger in some low-income and low-education subgroups than in more advantaged groups. This pattern is consistent with the possibility that AI-supported guidance and follow-up are particularly relevant to patients facing information and navigation barriers; however, subgroup differences in an observational sample do not demonstrate that AI narrowed causal health disparities. At the same time, the health equity-related interpretation should not be overstated. Associations were somewhat more consistent among participants with higher digital literacy, suggesting that the institutional availability of AI does not ensure equal individual use (6). Human assistance, simplified interfaces, and offline alternatives remain important for older adults with limited digital capabilities (18).

This finding speaks directly to current debates on AI and health inequity. d'Elia et al. (16) argued that AI in primary care can influence inequities both through direct care provision and through wider system-level effects. Sasseville et al. (5) further emphasized that bias mitigation in primary healthcare AI requires attention not only to algorithmic performance but also to protected attributes, population diversity, and implementation context. The present results support this cautious view. AI may help disadvantaged patients when it is used to simplify service navigation and improve provider-supported follow-up. But if AI services are delivered mainly through digital interfaces that require strong user capability, they may reproduce or even deepen existing digital exclusion. Therefore, the central policy issue is not simply whether primary care institutions have adopted AI systems, but whether these systems are accessible, understandable, and usable for older adults with weaker digital literacy.

The regional context should also be considered when interpreting the generalizability of these findings. Guangdong is one of China's more economically developed and digitally connected coastal provinces, and its major urban areas generally have relatively extensive broadband and 5G coverage, stronger technical capacity, and greater institutional resources for digital-health implementation (34, 35). However, substantial socioeconomic and digital disparities remain between major urban centers and less-developed, rural, and peripheral areas within the province. Compared with primary healthcare institutions in more rural or less-developed provinces, the participating institutions may have faced fewer constraints related to network connectivity, information-system maintenance, staff training, referral coordination, and technical support. Consequently, the institutional availability of AI and the 79.0% individual uptake rate observed in this study may not be readily reproduced in settings with weaker digital infrastructure, more limited primary care capacity, or lower patient digital literacy. The findings should therefore be interpreted as evidence from a relatively favorable but internally heterogeneous provincial context rather than as nationally representative estimates. Future research should include institutions from central and western China and resource-constrained rural areas to determine whether similar associations are observed under different socioeconomic and infrastructural conditions.

### 7.3 Health information acquisition and service accessibility as exploratory pathways

The third finding was that the observed data were statistically consistent with indirect associations involving health information acquisition and medical service accessibility. Both variables were associated with institutional AI availability and with the measured outcomes after covariate adjustment. Because all components were observed within a cross-sectional framework, this pattern does not show that AI availability preceded changes in information or accessibility or that those changes subsequently produced the outcomes. The information pathway is theoretically relevant for older chronic disease patients, who repeatedly decide how to interpret symptoms, manage daily behavior, return for follow-up, and seek referral (6). AI-supported reminders, referral advice, and personalized education may be associated with lower information barriers. The present cross-sectional analysis, however, does not establish that these functions caused improvements in self-management or health outcomes. The accessibility pathway is also theoretically relevant. AI-assisted triage, referral support, follow-up reminders, and risk warning may make care pathways more legible to older patients (11). In this study, perceived accessibility formed part of a statistically significant indirect association with hospitalization, but this finding is descriptive of the observed cross-sectional structure and should not be interpreted as causal prevention of hospitalization.

These findings have several practical implications. First, AI should be integrated into primary care workflows rather than treated as a stand-alone platform. The benefit of AI depends on whether doctors, nurses, chronic disease managers, and referral coordinators can use AI-generated information to support patient care. Second, AI-enabled services for older adults should preserve human assistance. For patients with low digital literacy, offline explanation, family doctor support, simplified interfaces, and assisted use may be necessary. Third, equity monitoring should become part of AI implementation. Primary care institutions should evaluate not only overall service efficiency but also whether AI benefits low-income, low-education, rural, older, or digitally disadvantaged patients. Finally, the findings must be interpreted with caution. The study used a non-randomized observational design, and some outcomes were based on self-reported measures. Although institutional matching, covariate adjustment, subgroup analysis, and robustness checks were used, residual confounding and reverse causality cannot be fully excluded. Future research should use longitudinal data, objective clinical indicators, and implementation-process evaluation to examine whether the observed associations persist over time and how AI-enabled primary care can be designed to promote equity more reliably.

## 8 Conclusion

This study examined associations between AI-enabled primary care, health outcomes, and health equity-related differences among older adults with chronic diseases in eight selected primary healthcare institutions in Guangdong Province. Institutional AI availability was associated with patient-reported chronic disease control, self-rated health, and hospitalization-related outcomes. Subgroup patterns were more pronounced in some low-income and low-education groups, whereas higher digital literacy was associated with more consistent individual benefit. Exploratory pathway analyses identified indirect cross-sectional associations involving health information acquisition and medical service accessibility. These findings do not establish a causal relationship or temporal sequence. They suggest that the potential equity relevance of AI-enabled primary care depends on staff support, inclusive implementation, service coordination, and accessible alternatives for patients with limited digital resources. Future multisite studies should combine patient-reported experiences with harmonized longitudinal clinical records to determine whether the observed subjective and cross-sectional associations correspond to sustained changes in disease control, healthcare utilization, and health equity over time.

## 9 Limitations and future research

Several limitations should be acknowledged. First, the non-randomized observational design cannot eliminate residual confounding. Institutional matching, covariate adjustment, alternative exposure definitions, and propensity score weighting address observed differences only. Family support and baseline self-management motivation were not measured and may be associated with both individual AI use and health outcomes. Second, institutional AI availability and individual actual use are not equivalent: 79.0% of participants in AI-enabled institutions reported use, while 21.0% did not. Individual-use estimates may therefore reflect selective uptake and are interpreted as supplementary associations. Third, the sample was drawn from eight institutions in Guangdong Province. Disease-type and urban-rural subgroup analyses are exploratory, and the findings may not generalize to regions with different primary care capacity, digital infrastructure, or socioeconomic conditions. Fourth, the pathway analysis was cross-sectional and cannot establish temporal precedence or a causal indirect pathway. Fifth, several key measures—including patient-reported chronic disease control, self-rated health, individual AI use, health information acquisition, medical service accessibility, and hospitalization experience—were based on participant reports. Although these measures capture patients' experiences and perceptions, they may be affected by recall bias, social desirability bias, and common-method variance. Objective blood pressure, fasting blood glucose, and HbA1c data were not sufficiently complete or harmonized across the eight institutions to support a reliable record-based sensitivity analysis. Future studies should link patient-reported measures with harmonized longitudinal clinical records, including repeated blood pressure and glucose measurements, HbA1c, medication adherence, referral completion, follow-up attendance, and verified hospitalization records. Sixth, qualitative coding used consensus comparison rather than a formal kappa statistic, and saturation was assessed thematically; these procedures are now reported explicitly. Future longitudinal, multisite studies should use harmonized objective clinical outcomes, richer measures of family and self-management context, and function-specific implementation data.

## Data Availability

The original contributions presented in the study are included in the article/Supplementary material, further inquiries can be directed to the corresponding author.
