# Supplementary material for: AI-assisted hierarchical primary care and health equity among older adults with chronic diseases: a matched observational mixed-methods study in China
Source: Front Public Health. 2026 Sep 22;14:1909288. doi: 10.3389/fpubh.2026.1909288 (PMC13638407; doi:10.3389/fpubh.2026.1909288)
Supplement: Supplementary file 1 [file Table_1.docx]

**Appendix: Patient Questionnaire and Medical Staff Interview Guide**

# Appendix A. Patient Questionnaire

Instructions for investigators: This questionnaire is intended for adults aged 60 years or above with physician-diagnosed chronic diseases who have used services in a participating primary healthcare institution within the past 6 months. Investigators should read each item aloud when necessary, use standardized wording, and avoid leading explanations. For participants with limited literacy, visual difficulty, or difficulty using electronic questionnaires, assisted administration is allowed, but responses should reflect the participant’s own views whenever possible.

Anonymous coding: Each participant should be assigned a unique study identification code at enrollment. The code should combine the institution code and a sequential participant number, for example PHC-A1-P001 for an AI-enabled institution or PHC-C1-P001 for a comparison institution. Direct identifiers such as name, phone number, national ID number, and medical record number should not be entered into the analytic dataset.

Response scale for perception items: 1 = strongly disagree, 2 = disagree, 3 = neutral/uncertain, 4 = agree, 5 = strongly agree. If an item is not applicable, select “Not applicable” where provided.

## A1. Screening, consent, and fieldwork information

| **Item** | **Question** | **Response / instruction** |
| --- | --- | --- |
| S0 | Institution code | □ PHC-A1 □ PHC-A2 □ PHC-A3 □ PHC-A4 □ PHC-C1 □ PHC-C2 □ PHC-C3 □ PHC-C4 |
| S1 | Participant study ID | __________ |
| S2 | Survey date | ____ / ____ / ______ |
| S3 | Age | ____ years. Eligible if age >= 60. |
| S4 | Has a physician ever diagnosed you with at least one chronic disease? | □ Yes □ No. If No, stop. |
| S5 | Have you used services in this primary healthcare institution within the past 6 months? | □ Yes □ No. If No, stop. |
| S6 | Can you understand the study information and provide informed consent independently or with appropriate assistance? | □ Yes □ No. If No, exclude. |
| S7 | Are you willing to participate in this study voluntarily? | □ Yes □ No. If No, stop. |
| S8 | Questionnaire administration mode | □ Self-administered paper □ Investigator-assisted paper □ Electronic questionnaire □ Investigator-assisted electronic questionnaire |

## A2. Sociodemographic characteristics

| **Item** | **Question** | **Response options** |
| --- | --- | --- |
| A1 | Sex | □ Male □ Female □ Other / prefer not to say |
| A2 | Marital status | □ Married □ Widowed □ Divorced/separated □ Never married |
| A3 | Living arrangement | □ Living alone □ With spouse □ With children □ With spouse and children □ Other |
| A4 | Highest education level | □ Primary school or below □ Junior high school □ Senior high/technical school □ College or above |
| A5 | Monthly household income | □ <3,000 RMB □ 3,000–5,999 RMB □ 6,000–9,999 RMB □ >=10,000 RMB □ Prefer not to say |
| A6 | Residence type | □ Urban community □ Rural/township area □ Other |
| A7 | Medical insurance type | □ Urban employee basic medical insurance □ Urban-rural resident basic medical insurance □ Commercial insurance □ Other □ None |
| A8 | Approximate distance from home to this primary healthcare institution | □ <1 km □ 1–3 km □ 3–5 km □ >5 km |
| A9 | Do you usually have someone who can help you seek medical care when needed? | □ Yes, family member □ Yes, friend/neighbour □ Yes, community worker □ No regular helper |

## A3. Health status and chronic disease profile

| **Item** | **Question** | **Response options** |
| --- | --- | --- |
| B1 | Which chronic diseases have you been diagnosed with? Select all that apply. | □ Hypertension □ Diabetes □ Cardiovascular disease □ Chronic respiratory disease □ Stroke sequelae □ Chronic kidney disease □ Other: ____ |
| B2 | Duration of the main chronic disease | □ <1 year □ 1–4 years □ 5–9 years □ >=10 years |
| B3 | Number of physician-diagnosed chronic diseases | □ 1 □ 2 □ 3 or more |
| B4 | How would you rate your current health status? | 1 = very poor, 2 = poor, 3 = fair, 4 = good, 5 = very good |
| B5 | During the past 12 months, how many times were you hospitalized because of chronic disease or related complications? | ____ times |
| B6 | During the past 12 months, did you have any emergency visit related to chronic disease? | □ Yes □ No □ Not sure |
| B7 | During the past 6 months, did you have any acute worsening of chronic disease symptoms? | □ Yes □ No □ Not sure |

## A4. Digital literacy

| **Item** | **Question** | **Response** |
| --- | --- | --- |
| C1 | I can use a smartphone independently. | 1–5 Likert scale |
| C2 | I can use WeChat or similar applications to communicate with others. | 1–5 Likert scale |
| C3 | I can make a medical appointment online, either independently or with limited assistance. | 1–5 Likert scale |
| C4 | I can read and understand health information from mobile phones or the internet. | 1–5 Likert scale |
| C5 | I can use online consultation, internet hospitals, or health apps when needed. | 1–5 Likert scale |
| C6 | When I encounter digital health service problems, I can find someone to help me. | 1–5 Likert scale |

## A5. AI exposure and use

Investigator note: D0 should be completed according to institutional records. D1–D8 should be answered by the patient. For comparison institutions without AI-assisted hierarchical diagnosis and treatment, D1–D8 may be coded as “No” or “Not applicable” according to the response options.

| **Item** | **Question** | **Response** |
| --- | --- | --- |
| D0 | Institutional AI access status | Investigator-completed: □ AI-enabled institution □ Comparison institution |
| D1 | Has this institution provided intelligent guidance, AI-assisted triage, AI follow-up reminders, AI risk warning, AI referral support, or AI-related health consultation to you? | □ Yes □ No □ Not sure |
| D2 | Which AI-assisted services have you used or received? Select all that apply. | □ Intelligent guidance □ AI-assisted triage □ AI follow-up reminder □ AI risk warning □ AI-supported referral advice □ AI-related consultation explained by staff □ None □ Not sure |
| D3 | In the past 6 months, how often have you used or received AI-assisted services? | □ Never □ 1–2 times □ 3–5 times □ >5 times □ Not sure |
| D4 | How did you mainly use or receive AI-assisted services? | □ Independently through phone/device □ With help from medical staff □ With help from family □ Indirectly through doctor/nurse explanation □ Not applicable |
| D5 | The AI-assisted service helped me understand where I should seek care. | 1–5 Likert scale / □ Not applicable |
| D6 | The AI-assisted service helped me receive follow-up reminders or health management advice. | 1–5 Likert scale / □ Not applicable |
| D7 | The AI-assisted service helped me understand whether referral to a higher-level hospital was needed. | 1–5 Likert scale / □ Not applicable |
| D8 | I trust AI-assisted advice when it is explained or confirmed by medical staff. | 1–5 Likert scale / □ Not applicable |

## A6. Health information acquisition

| **Item** | **Question** | **Response** |
| --- | --- | --- |
| E1 | I know how to manage my chronic disease in daily life. | 1–5 Likert scale |
| E2 | I can obtain timely advice when my symptoms change. | 1–5 Likert scale |
| E3 | I know when I should visit a primary healthcare institution and when I should go to a higher-level hospital. | 1–5 Likert scale |
| E4 | I receive personalized reminders or suggestions about medication, follow-up, diet, exercise, or risk warning. | 1–5 Likert scale |
| E5 | The health information I receive from this institution is easy to understand. | 1–5 Likert scale |
| E6 | Medical staff help me understand digital or AI-generated health information when needed. | 1–5 Likert scale |

## A7. Medical service accessibility and health outcomes

| **Item** | **Question** | **Response** |
| --- | --- | --- |
| F1 | When you feel unwell because of chronic disease, do you usually first visit a primary healthcare institution? | □ Yes □ No □ It depends |
| F2 | Average waiting time for routine chronic disease consultation | □ <15 minutes □ 15–30 minutes □ 31–60 minutes □ >60 minutes |
| F3 | In the past 6 months, did you receive regular follow-up from primary care staff? | □ Yes □ No □ Not sure |
| F4 | If referral was needed, the referral process was clear and convenient. | 1–5 Likert scale / □ Not applicable |
| F5 | It is convenient for me to obtain chronic disease management services from this institution. | 1–5 Likert scale |
| F6 | In the past 3 months, has your blood pressure or blood glucose been controlled within the target range? | □ Yes □ No □ Not applicable □ Not sure |
| F7 | In the past 12 months, did you have any emergency visit or hospitalization related to chronic disease? | □ Yes □ No □ Not sure |
| F8 | Overall, I am satisfied with the chronic disease management services provided by this institution. | 1–5 Likert scale |

## A8. Optional clinical record abstraction form

This section should be completed only when the participant has provided additional consent for extracting relevant clinical indicators from institutional records. Record only de-identified clinical data linked by the study ID.

| **Item** | **Indicator** | **Record** |
| --- | --- | --- |
| R1 | Latest systolic / diastolic blood pressure | ____ / ____ mmHg; date: ____ |
| R2 | Latest fasting blood glucose | ____ mmol/L; date: ____ |
| R3 | Latest HbA1c | ____ %; date: ____ |
| R4 | Number of primary care follow-up records in past 6 months | ____ times |
| R5 | Referral record in past 12 months | □ Yes □ No; reason: ____ |
| R6 | Hospitalization record in past 12 months | □ Yes □ No; number of hospitalizations: ____ |
| R7 | Data source | □ Chronic disease management record □ Follow-up record □ Electronic health record □ Other: ____ |

# Appendix B. Medical Staff Interview Guide

Target participants: general practitioners, nurses, public health physicians, chronic disease managers, family doctor team members, triage staff, referral coordinators, AI system operators, and institutional administrators involved in primary chronic disease services.

Interview duration: approximately 30–45 minutes. Interviews may be audio-recorded after written consent. The interviewer should encourage participants to describe concrete examples rather than general impressions.

## B1. Interview record and anonymous coding

| **Item** | **Field** | **Response / instruction** |
| --- | --- | --- |
| I1 | Institution code | □ PHC-A1 □ PHC-A2 □ PHC-A3 □ PHC-A4 □ PHC-C1 □ PHC-C2 □ PHC-C3 □ PHC-C4 |
| I2 | Staff interview code | __________ Example: PHC-A1-GP03, PHC-A2-Nurse07, PHC-A4-Manager02 |
| I3 | Interview date | ____ / ____ / ______ |
| I4 | Participant role | □ General practitioner □ Nurse □ Public health physician □ Chronic disease manager □ Family doctor team member □ Triage/referral staff □ AI system operator □ Administrator □ Other |
| I5 | Audio-recording consent | □ Yes □ No |

## B2. Opening script

Thank you for participating in this interview. This study aims to understand how AI-assisted hierarchical diagnosis and treatment is used in primary healthcare and how it may influence chronic disease management for older adults. There are no right or wrong answers. We are interested in your actual work experience. Your identity and institution will be anonymized in all research outputs, and you may stop the interview at any time.

## B3. Core interview questions

| **No.** | **Theme** | **Question** |
| --- | --- | --- |
| 1 | Role and work context | Could you briefly describe your position and your responsibilities in chronic disease management or primary care services? |
| 2 | AI system description | What AI-assisted systems or digital tools are currently used in your institution? Please describe the system name, deployment time, main functions, and target users where possible. |
| 3 | Workflow integration | How are AI-assisted tools integrated into daily service processes? At which stage are they used most often: guidance, triage, follow-up, referral, risk warning, or decision support? |
| 4 | Routine follow-up and patient guidance | Do AI tools help staff conduct routine follow-up or provide clearer guidance for older chronic disease patients? Please provide concrete examples. |
| 5 | Information acquisition | Do AI tools help older patients better understand disease management, medication, follow-up, warning signs, or referral pathways? Please provide examples. |
| 6 | Service accessibility | Do AI tools influence waiting time, first-contact care, referral convenience, follow-up frequency, or continuity of care? Please provide examples. |
| 7 | Low digital literacy and staff-mediated use | How do patients with low digital literacy use AI-enabled services? Do medical staff or family members usually need to help them? |
| 8 | Equity and exclusion | Which older chronic disease patients benefit most from AI-enabled services? Are there patients who may be excluded or benefit less? Why? |
| 9 | Provider acceptance | How do medical staff perceive AI recommendations? Are they trusted, modified, ignored, or used only as reference? |
| 10 | Actionability of AI recommendations | When the system generates a reminder, risk alert, triage suggestion, or referral recommendation, who is responsible for acting on it? What makes the recommendation actionable or not actionable? |
| 11 | Risks and governance | What risks or problems have you observed, such as inaccurate recommendations, privacy concerns, workload changes, responsibility ambiguity, or patient misunderstanding? |
| 12 | Improvement suggestions | What institutional support is needed to make AI-assisted hierarchical diagnosis and treatment more useful, safe, and equitable for older chronic disease patients? |

## B4. Closing questions

| **Item** | **Question** |
| --- | --- |
| C1 | Is there anything else you think is important for understanding AI-assisted primary care for older chronic disease patients? |
| C2 | Would you be willing to be contacted again if clarification is needed? □ Yes □ No |

# Appendix C. Investigator Fieldwork and Data Management Checklist

| **Stage** | **Checklist** |
| --- | --- |
| Before fieldwork | Confirm institutional permission; verify AI deployment status; prepare consent forms; prepare anonymous ID list; print large-font questionnaires; test electronic survey system; train investigators. |
| During recruitment | Apply inclusion/exclusion criteria; avoid interrupting clinical care; explain voluntary participation; obtain informed consent; assign unique study ID. |
| During survey | Use standardized wording; provide neutral assistance; ensure responses reflect the participant’s own views; check completion before the participant leaves. |
| After survey | Check missing key variables; identify duplicate records; check logical consistency; separate consent forms from questionnaires; store paper questionnaires securely. |
| Clinical record abstraction | Extract clinical indicators only after additional consent; use study ID rather than direct identifiers; record data source and date of measurement. |
| Interview management | Obtain recording consent; use anonymous staff code; remove names and institution identifiers during transcription; store audio files securely. |
| Data entry and cleaning | Use double data entry or automated logic checks; document exclusions; record missingness; prepare a de-identified analytic dataset. |

# Appendix D. Institution-level AI deployment record

This form should be completed by the research team based on institutional documents, system screenshots where permitted, and confirmation from local coordinators. It is used to distinguish institutional AI access from patient-level AI use.

| **Item** | **Field** | **Record** |
| --- | --- | --- |
| D-AI1 | Institution code | PHC-A1 / PHC-A2 / PHC-A3 / PHC-A4 / PHC-C1 / PHC-C2 / PHC-C3 / PHC-C4 |
| D-AI2 | AI deployment status | □ AI-enabled □ Comparison institution without structured AI-assisted hierarchical diagnosis and treatment |
| D-AI3 | AI system name or platform | __________ |
| D-AI4 | Deployment date | ____ / ____ / ______ |
| D-AI5 | Functions in routine use | □ Intelligent guidance □ AI-assisted triage □ Follow-up reminder □ Risk warning □ Referral support □ Decision support □ Other: ____ |
| D-AI6 | Main users | □ Patients □ Doctors □ Nurses □ Chronic disease managers □ Administrators □ Other |
| D-AI7 | Whether system outputs are linked to staff workflow | □ Yes □ Partly □ No; explanation: ____ |
| D-AI8 | Whether staff training was provided | □ Yes □ No □ Not sure; date or description: ____ |
